# pK205R targets the proximal element of IFN-I signaling pathway to assist African swine fever virus to escape host innate immunity at the early stage of infection

**DOI:** 10.1371/journal.ppat.1012613

**Published:** 2024-10-15

**Authors:** Zhao Huang, Cuiying Kong, WenBo Zhang, Jianyi You, Chenyang Gao, Jiangnan Yi, Zhanzhuo Mai, Xiongnan Chen, Pei Zhou, Lang Gong, Guihong Zhang, Heng Wang

**Affiliations:** 1 Key Laboratory of Zoonosis Prevention and Control of Guangdong Province, College of Veterinary Medicine, South China Agricultural University, Guangzhou, China; 2 African Swine Fever Regional Laboratory of China (Guangzhou), Guangzhou, China; 3 Research Center for African Swine Fever Prevention and Control, South China Agricultural University, Guangzhou, China; 4 Maoming Branch, Guangdong Laboratory for Lingnan Modern Agriculture, Guangdong, China; 5 Key Laboratory of Animal Vaccine Development, Ministry of Agriculture and Rural Affairs, Guangzhou, China; University of Southern California, UNITED STATES OF AMERICA

## Abstract

African swine fever virus (ASFV) is a nuclear cytoplasmic large DNA virus (NCLDV) that causes devastating hemorrhagic diseases in domestic pigs and wild boars, seriously threatening the development of the global pig industry. IFN-I plays an important role in the body’s antiviral response. Similar to other DNA viruses, ASFV has evolved a variety of immune escape strategies to antagonize IFN-I signaling and maintain its proliferation. In this study, we showed that the ASFV early protein pK205R strongly inhibited interferon-stimulated genes (ISGs) as well as the promoter activity of IFN-stimulated regulatory elements (ISREs). Mechanistically, pK205R interacted with the intracellular domains of IFNAR1 and IFNAR2, thereby inhibiting the interaction of IFNAR1/2 with JAK1 and TYK2 and hindering the phosphorylation and nuclear translocation of STATs. Subsequently, we generated a recombinant strain of the ASFV-pK205R point mutation, ASFV-pK205R^7PM^. Notably, we detected higher levels of ISGs in porcine alveolar macrophages (PAMs) than in the parental strain during the early stages of ASFV-pK205R^7PM^ infection. Moreover, ASFV-pK205R^7PM^ attenuated the inhibitory effect on IFN-I signaling. In conclusion, we identified a new ASFV immunosuppressive protein that increases our understanding of ASFV immune escape mechanisms.

## Author summary

African Swine fever virus (ASFV) causes a deadly swine disease that originated in Africa and has since spread to Europe, Asia, and Oceania, severely impacting the global pig industry. Although ASFV protein functions have been extensively studied, many remain unknown. pK205R is abundantly expressed during the early stages of ASFV infection and participates in viral replication during the late stages of infection. Our research reveals how pK205R inhibits IFN-I signaling by interacting with IFNAR1 and IFNAR2, blocking downstream signaling. These results improve our understanding of the immune escape process of ASFV, which may have implications for future vaccine development.

## Introduction

African swine fever is an acute hemorrhagic infectious disease in pigs caused by the African swine fever virus (ASFV), which is currently present in many countries in Eastern Europe, the Russian Federation, and Southeast Asia, severely impacting the pig industry. At present, ASFV has been recombined and mutated, including weak strains that cause persistent infection in pigs and recombinant strains of genotypes I and II that are more virulent [1–3]. Currently, there is no safe and effective commercial vaccine or therapeutic drug, making ASF prevention and control extremely challenging [4].

Type I interferon (IFN-I) is a pleiotropic multifunctional protein involved in early immune responses with immunomodulatory and highly effective antiviral functions [5]. The Janus kinase/signal transducer and activator of transcription (JAK/STAT) pathway, activated by IFN-I, is crucial for establishing an antiviral state [6,7]. IFN-I binds to IFN-I receptor (IFNAR) 1 and IFNAR2 to initiate signaling [8]. These receptors are class II helical cytokine receptor transmembrane proteins [9,10], with the extracellular domain (ECD) binding to IFN-I [11] and the intracellular domain (ICD) interacting with JAK1 and TYK2 kinases. Ligand binding leads to TYK2 and JAK1 cross-phosphorylation [8]. This recruits STAT1 and STAT2 to the receptor complex, where activated JAKs phosphorylate them. The phosphorylated STAT1/2 and IFN regulatory factor 9 (IRF9) form the trimeric complex interferon-stimulated gene factor 3 (ISGF3), which translocates to the nucleus to initiate transcription of interferon-stimulated genes (ISGs) [12,13] and antiviral functions [14].

ASFV belongs to a group of nucleocytoplasmic large DNA viruses (NCLDVs) with a genome consisting of 170–190 kbp double-stranded DNA molecules, containing 151–167 open reading frames (ORFS) [15]. Single-cell sequencing results showed that the ISG expression in ASFV-infected cells was significantly lower than that in uninfected cells, and the cellular ISG score was significantly negatively correlated with viral load, indicating that the antagonism of IFN-I antiviral response by ASFV is critical for promoting efficient viral replication [16]. ASFV employs various proteins and mechanisms to antagonize IFN signaling [17,18], indicating a well-developed immune escape system. However, the functions of some immunosuppressive proteins remain unknown.K205R is an early gene of ASFV encoding a non-structural protein pK205R with 205 amino acids. pK205R is abundantly expressed in the early stage of ASFV infection, diffusely expressed in the cytoplasm, and enriched in virus factories in the middle and late stages of infection [19]. However, pK205R has not been shown to inhibit IFN-I-mediated innate immune responses. Herein, we found that pK205R targeted IFNAR to inhibit the phosphorylation and nuclear translocation of STATs, thereby inhibiting IFN-I signaling.

## Results

### pK205R antagonism of IFN-I signal transduction promotes viral replication

**Fig 1:**
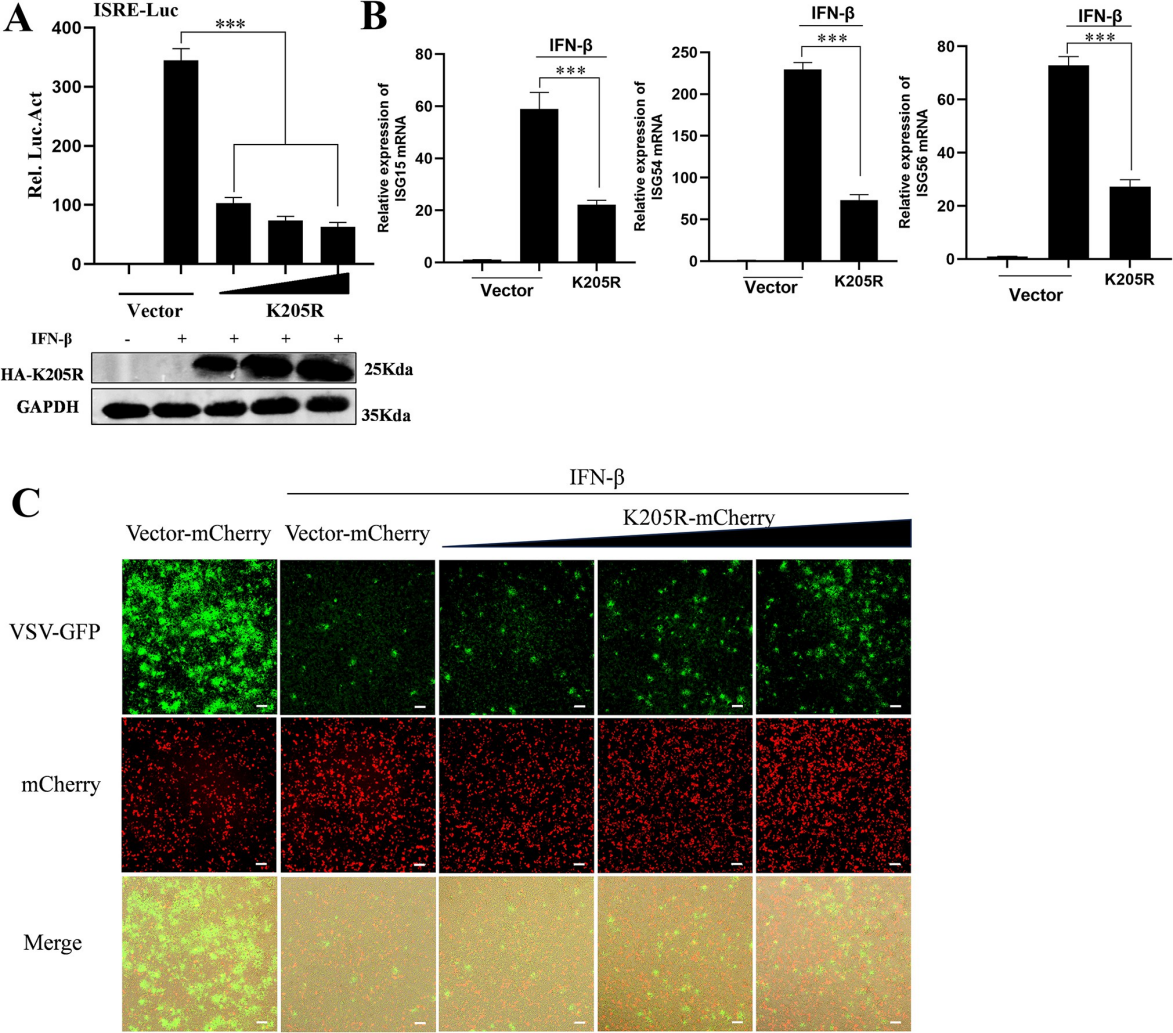
ASFV pK205R antagonizes IFN-I signaling. (A) HEK293T cells cultured in 24-well plates were transfected with ASFV K205R expression plasmid or empty vector (100 ng, 200 ng, or 400 ng), along with PRL-TK (25 ng) and ISRE-Luc (125 ng). At 24 hpt, cells were treated with 1,000 U/mL IFN-β for 8 h and analyzed using a dual-luciferase reporter assay. (B) HEK293T cells cultured in 24-well plates were transfected with ASFV K205R expression plasmid or empty vector (500 ng). After 24 h, cells were treated with 1,000 U/mL IFN-β for 8 h and analyzed through qPCR. (C) HEK293T cells were transfected with the pK205R-mCherry expression plasmid (200 ng, 400 ng, or 800 ng) or empty vector. At 16 hpt, cells were stimulated with 1,000 U/ml IFN-β and inoculated with VSV-GFP at an MOI of 0.1. At 24 hpt. Cells were cultured for an additional 24 h. GFP and mCherry signals were observed under a microscope (scale bar: 100 μm). *, P < 0.05; * *, P < 0.01; ***, P < 0.001.

Our previous study found that ASFV pK205R strongly inhibited IFN-I-activated ISRE promoter activity [20]. To further explore the role of ASFV pK205R in regulating IFN-I signaling, we co-transfected the pK205R plasmid with pRL-TK and ISRE-luc at different doses and set up a control group transfected with pCAGGS-HA. At 24 h post-transfection (hpt), cells were treated with IFN-β for 8 h, and the results were detected using a dual-luciferase reporter gene system. pK205R significantly reduced IFN-I-activated ISRE promoter activity, and ISRE promoter activity was inversely related to pK205R dose (Fig 1A). The mRNA levels of ISGs are important indicators of IFN-I antiviral activity. To explore the effect of pK205R on IFN-I-activated ISG transcription, we examined the effect of pK205R on ISG15, ISG54, and ISG56 mRNA levels using qPCR. As shown in Fig 1B, the mRNA levels of ISG15, ISG56, and ISG54 in the pK205R group were significantly lower than those in the group transfected with empty IFN-β stimulation. Next, we investigated whether pK205R could inhibit the antiviral activity of IFN-I using vesicular stomatitis virus (VSV), which is highly sensitive to IFN-I [21]. The results are shown in Fig 1C. As expected, VSV-GFP replication was almost completely inhibited in cells pretreated with IFN-I. However, pK205R promoted VSV-GFP replication, and the proliferation of VSV-GFP was positively correlated with the dose of pK205R, indicating that pK205R weakened the inhibitory effect of IFN-I on VSV-GFP proliferation and inhibited the antiviral effect of IFN-β. Collectively, our results suggest that pK205R can promote viral replication by inhibiting IFN-I signaling.

### pK205R reduces JAK and STAT2 phosphorylation, inhibiting STAT1 nuclear localization

**Fig 2:**
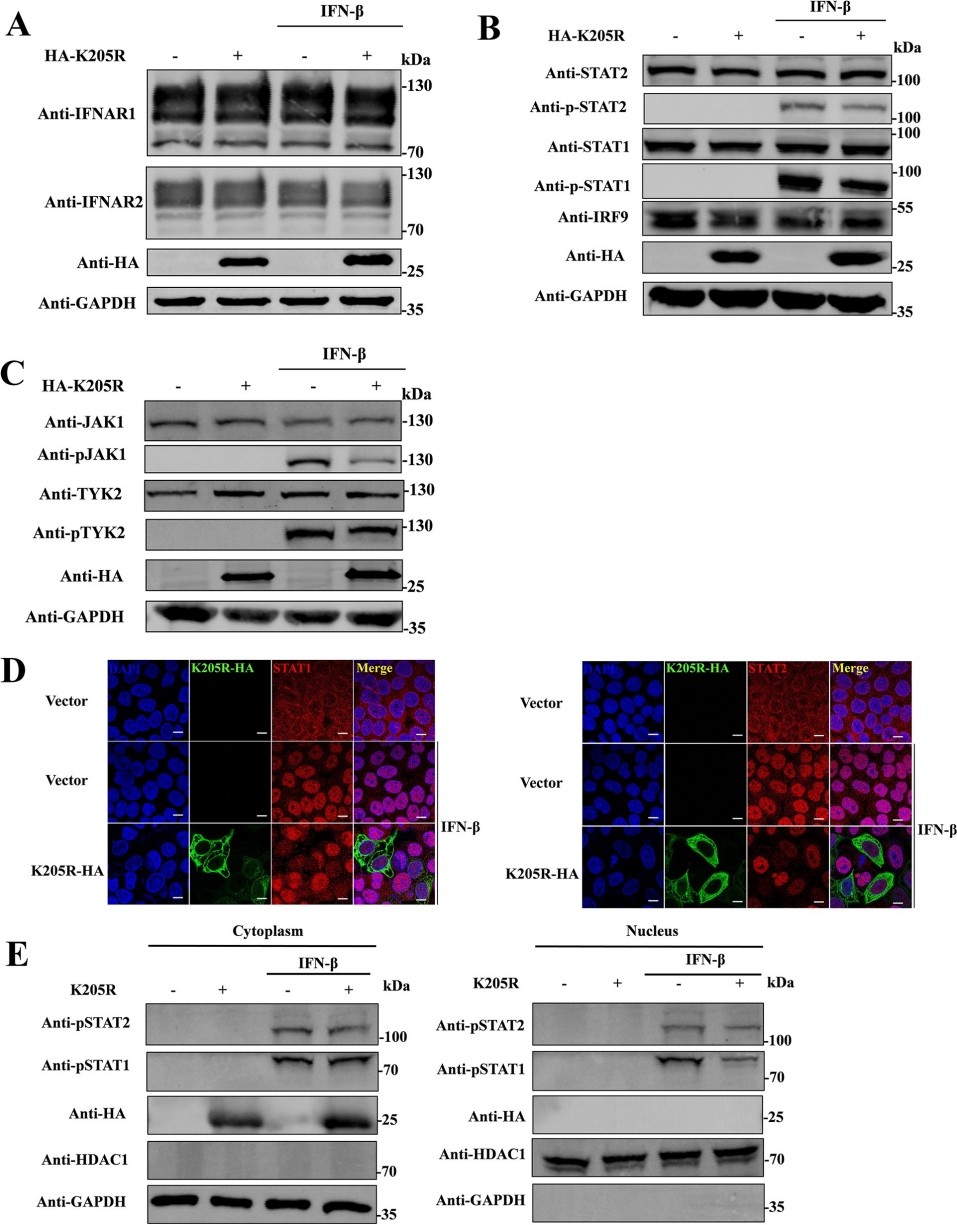
pK205R inhibits the JAK/STAT signal transduction pathway. (A–C) HEK293T cells cultured in 12-well plates were transfected with pK205R expression plasmid (1 μg). At 24 hpt, cells were stimulated with or without IFN-β (1,000 U/mL) for 2 h. Expression of the indicated proteins was determined via western blotting. (D) pK205R (2.5 μg/ well) was transfected into HeLa cells in glass-bottom dishes. At 24 hpt, cells were stimulated with IFN-β (1,000 U/mL) for 2 h. Cells were stained with anti-STAT1/2 (red), and nuclei were stained with DAPI. Subcellular localization of the indicated proteins was analyzed by confocal microscopy (scale bar: 10 μm). (E) pK205R (2.5 μg/ well) was transfected into HEK293T cells in 6-well plates. At 24 hpt, the cells were stimulated with IFN-β (1,000 U/mL) for 2 h. Cytoplasmic and nuclear proteins were extracted from the cells using the NE-PER nuclear and cytoplasmic extraction kit. Levels of p-STAT1/2 in nuclear and cytoplasmic compartments was detected through western blotting. GAPDH and heat shock protein HDAC1 were used as cytoplasmic and nuclear markers, respectively.

To explore the key points in the inhibition of JAK/STAT signaling by pK205R, such as JAK and STAT phosphorylation and STAT nuclear translocation, we transfected pK205R into HEK293T cells and detected the expression of JAK/STAT pathway proteins at 24 hpt. As shown in Fig 2A–2C, the protein levels of IFNAR1, IFNAR2, JAK1, TYK2, STAT1, STAT2, and IRF9 did not considerably change in the pK205R transfected group, regardless of whether the cells were treated with IFN-β, compared with the Mock group (S1A–S1C Fig). As expected, IFN-β treatment induced the phosphorylation of JAK1, TYK2, STAT1, and STAT2. Importantly, pK205R greatly inhibited IFN-I-activated phosphorylation of JAKs and STATs (Figs 2B, 2C, S1B and S1C). Nuclear translocation of STATs is a key process in IFN-I signaling, and phosphorylation of STATs is necessary for nuclear translocation. Therefore, blocking STAT phosphorylation would theoretically lead to the failure of STAT nuclear translocation. To further elucidate the mechanism of action of pK205R against IFN-I, we investigated the effect of pK205R on STAT1 and STAT2 nuclear translocation using laser confocal microscopy. As shown in Figs 2D and S1D, in the absence of IFN-β, STAT1 and STAT2 was indicated by red fluorescence and was distributed throughout the cytoplasm and nucleus. Following IFN-β treatment, STAT1 and STAT2 was concentrated in the nucleus. Nuclear accumulation of STAT1 and STAT2 was greatly reduced in pK205R expressing cells. This phenomenon was confirmed via a nuclear separation assay; pK205R caused a significant reduction in the accumulation of p-STAT1 and p-STAT2 in the nucleus (Figs 2E and S1E). Therefore, pK205R inhibited the phosphorylation of JAKs and STATs, as well as the nuclear translocation of STAT1, suggesting it may target proteins upstream of JAK phosphorylation to inhibit the IFN-I-mediated JAK/STAT signaling pathway.

### pK205R interacts with both IFNAR1 and IFNAR2

**Fig 3:**
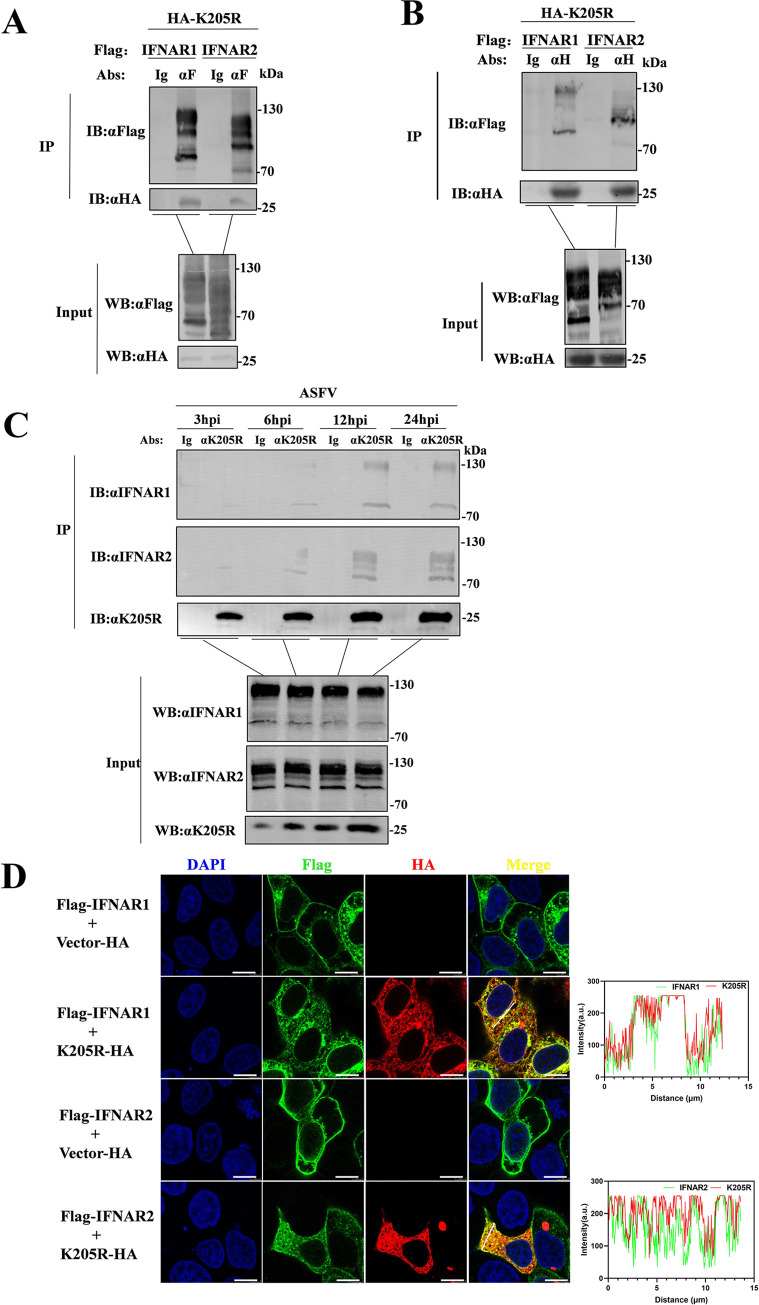
pK205R interacts with both IFNAR1 and IFNAR2. (A, B) HEK293T cells were transfected with pK205R (5 μg) and either IFNAR1 or IFNAR2 (5 μg) in 100-mm petri dishes. At 24 hpt, cell lysates were subjected to Co-IP using HA and FLAG antibodies, followed by immunoblotting with FLAG and HA antibodies, respectively. (C) PAMs were infectted with ASFV-WT or ASFV-pK205R7PM in 100-mm dishes. Co-IP of cell lysates was performed using mouse pK205R antibodies at 3, 6, 12, and 24 hpi, followed by protein immunoblotting using rabbit pK205R, IFNAR1, and IFNAR2 antibodies. (D) pK205R (1 μg) and either IFNAR1 or IFNAR2 (1 μg) were co-transfected into HeLa cells in glass-bottom dishes. At 24 hpt, the cells were stained with anti-FLAG (green) and anti-HA (red) antibodies, and the nucleus was stained with DAPI (blue). Colocalization of the indicated proteins was analyzed via confocal microscopy (scale bar: 10 μm). For the fluorescence intensity analysis, red and green wavy lines indicate fluorescence of the corresponding color. The ordinate represents the gray value of the fluorescence channel, and the abscissa represents the relative position of the lines in the merged plot.

To explore the mechanism by which pK205R downregulates the phosphorylation of STAT1, STAT2, JAK1, and TYK2, a co-immunoprecipitation (Co-IP) assay was performed. The results showed that pK205R interacted with IFNAR1 and IFNAR2 (Fig 3A and 3B) but not with JAK1, TYK2, STAT1, STAT2, or IRF9 (S2A Fig). In addition, we assessed the interaction of pK205R with endogenous IFNAR1 and IFNAR2 at various time points after ASFV infection The interaction between pK205R with IFNAR1 and IFANR2 increased as the duration of ASFV infection progressed (Fig 3C). To further confirm the interaction between pK205R and IFNAR1/IFNAR2, we used laser confocal microscopy to examine the subcellular localization of pK205R with exogenous IFNAR1 and IFNAR2. The results indicated that pK205R is primarily expressed in the cytoplasm (S2B Fig). In cells co-expressing pK205R and IFNAR1, pK205R strongly co-localized with IFNAR1, with fluorescence intensity analyzed using ImageJ software, as shown by the white indicator lines (Fig 3D). Our findings suggest that pK205R interacts with both IFNAR1 and IFNAR2.

### pK205R inhibits the interaction of IFNAR1 with TYK2 and IFNAR2 with JAK1

**Fig 4:**
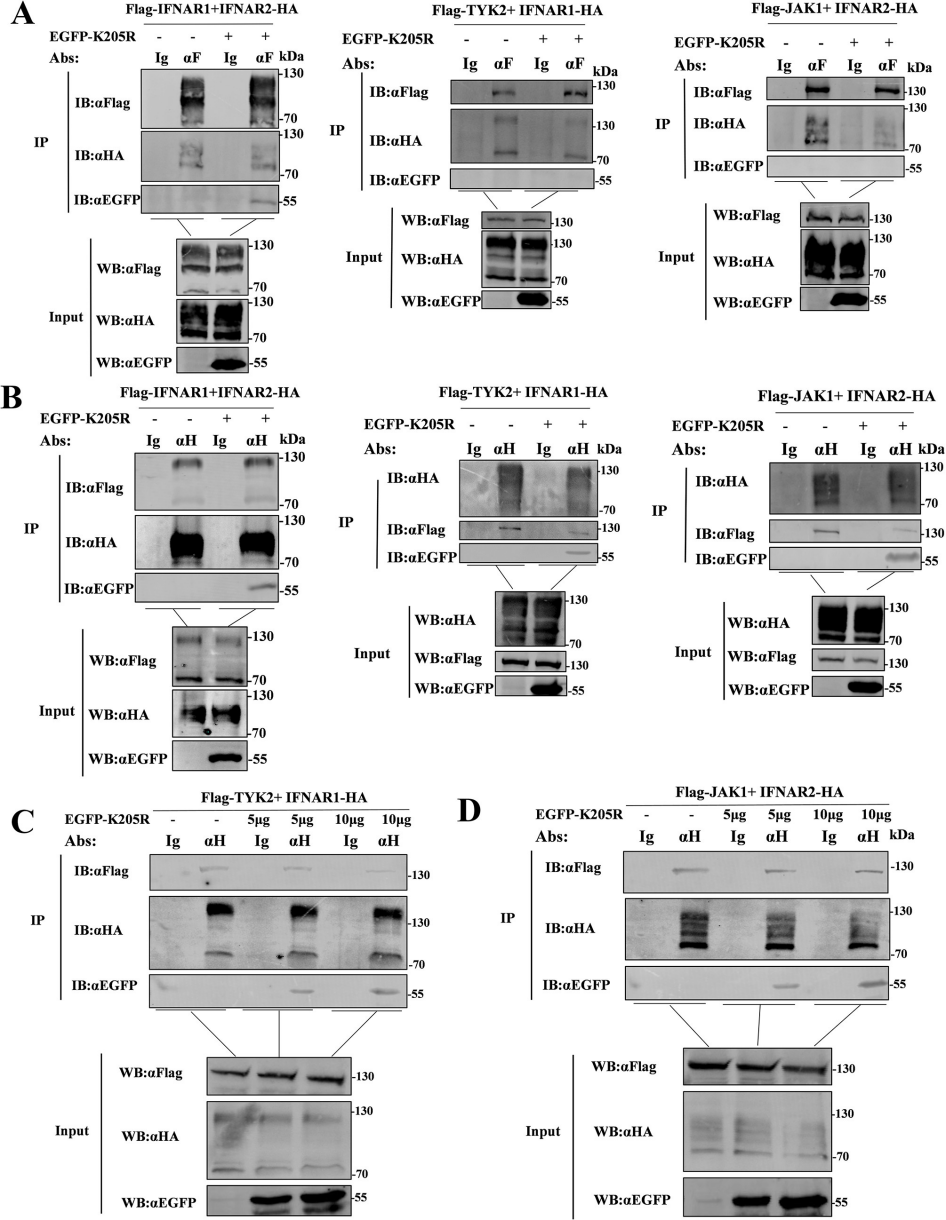
pK205R inhibits the binding of IFNAR1 with TYK2 and IFNAR2 with JAK1. (A, B) IFNAR1 (5 μg) and IFNAR2 (5 μg), or IFNAR1 (5 μg) and TYK2 (5 μg), or JAK1 (5 μg) and IFNAR2 (5 μg) were co-transfected into HEK293T cells with pK205R-EGFP (5 μg) or an empty vector in 100-mm dishes. Co-IP of cell lysates was performed using at 24 hpt using HA and FLAG antibodies, followed by immunoblotting with FLAG, HA, and EGFP antibodies. (C, D) IFNAR1 (5 μg) and TYK2 (5 μg) or JAK1 (5 μg) and IFNAR2 (5 μg) were co-transfected into HEK293T cells with varying doses of pK205R-EGFP (5 μg, 10 μg) or empty vector in 100 mm dishes. Co-IP of cell lysates was performed at 24 hpt using HA and FLAG antibodies, followed by immunoblotting using FLAG, HA, and EGFP antibodies.

Since pK205R interacts with IFNAR and inhibits the phosphorylation of JAKs and STATs, and TYK2/JAK1 must bind to IFNAR1/2 to function as phosphokinases [22], we hypothesized that pK205R antagonizes the binding of IFNAR1 to IFNAR2 or IFNAR to JAKs. To test our hypothesis, pK205R was co-transfected with IFNAR1/2 and TYK2/JAK1 into HEK293T cells and treated with IFN-β for 2 h at 24 hpt. The protein lysates were then used in Co-IP and western blot experiments. The results showed that pK205R did not suppress the interaction between IFNAR1 and IFNAR2. Notably, pK205R strongly inhibited the interaction of IFNAR2 with JAK1 and IFNAR1 with TYK2 (Fig 4A and 4B). In order to strengthen our conclusion, we transfected pK205R with different doses to detect its effect on the interaction between IFNAR1 and TYK2, IFNAR2 and JAK1, respectively. As shown in Fig 4C, increasing doses of pK205R led to a gradual decrease in the precipitation of TYK2 and JAK1 by IFNAR1 and IFNAR2. These results suggest that pK205R inhibits the interaction between IFNAR1 and TYK2, and IFNAR2 and JAK1, in a dose-dependent manner. Therefore, pK205R inhibits the phosphorylation of JAKs and STATs by blocking the interaction of IFNARs with JAKs.

### pK205R mainly interacts with the ICD of IFNAR1 and IFNAR2

**Fig 5:**
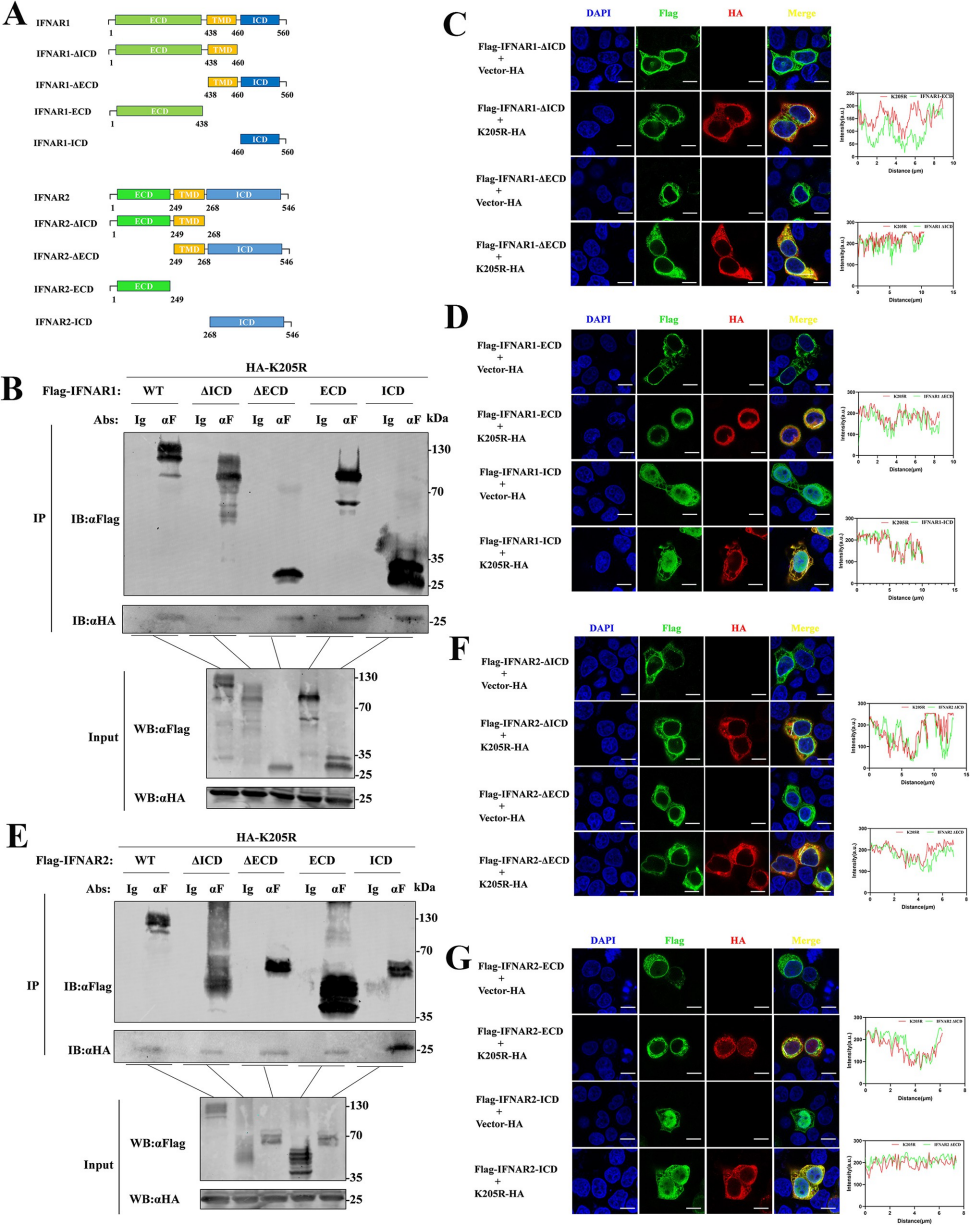
Identification of key domains for pK205R interaction with IFNARs. (A) Schematic representation of the construction of truncated mutant plasmids for IFNAR1 and IFNAR2. (B, E) HEK293T cells in 100-mm dishes were co-transfected with pK205R (5 μg) and either IFNAR1/2 (5 μg), IFNAR1/2-ΔICD (5 μg), IFNAR1/2-ΔECD (5 μg), IFNAR1/2-ICD (5 μg), or IFNAR1/2-ECD (5 μg). At 24 hpt, cell lysates were subjected to Co-IP using HA and FLAG antibodies, followed by immunoblotting with FLAG and HA antibodies. (C, D, F, G) HeLa cells in glass-bottom dishes were transfected with pK205R (1 μg) and either IFNAR1/2-ΔICD (1 μg), IFNAR1/2-ΔECD (1 μg), IFNAR1/2-ICD (1 μg), or IFNAR1/2-ECD (1 μg), alone or in combination. At 24 hpt, cells were stained with anti-FLAG (green) and anti-HA (red) antibodies, and the nucleus was stained with DAPI (blue). Colocalization of the indicated proteins was analyzed by confocal microscopy (scale bar: 10 μm). For the fluorescence intensity analysis, red and green wavy lines indicate fluorescence of the corresponding color. The ordinate represents the gray value of the fluorescence channel, and the abscissa represents the relative position of the lines in the merged plot.

To further elucidate the mechanism by which pK205R inhibits the interaction between IFNARs and JAKs, we created truncated mutants of IFNAR1 and IFNAR2 (Fig 5A) and investigated the key interaction domains of pK205R using Co-IP and laser confocal microscopy. pK205R interacted and co-localized with all four truncated mutants of IFNAR1, with the interaction being the most pronounced between the ICD domain of pK205R and IFNAR1. (Fig 5B–5D). Similarly, pK205R interacted and co-localized with all truncated mutants of IFNAR2, showing a stronger interaction with mutants containing the ICD (Fig 5E–5G). These results indicate that pK205R primarily interacts with the ICD of IFNAR2. Interestingly, we found that the ICD of IFNAR1 and IFNAR2 were internalized into the nucleus when the TM domain was absent (Fig 5D and 5G), indicating that the TM domain is essential for maintaining IFNAR1 and IFNAR2 ICD distribution in the cytoplasm.

### Deletion of the domain of pK205R alone had no effect on IFN-I signaling

**Fig 6:**
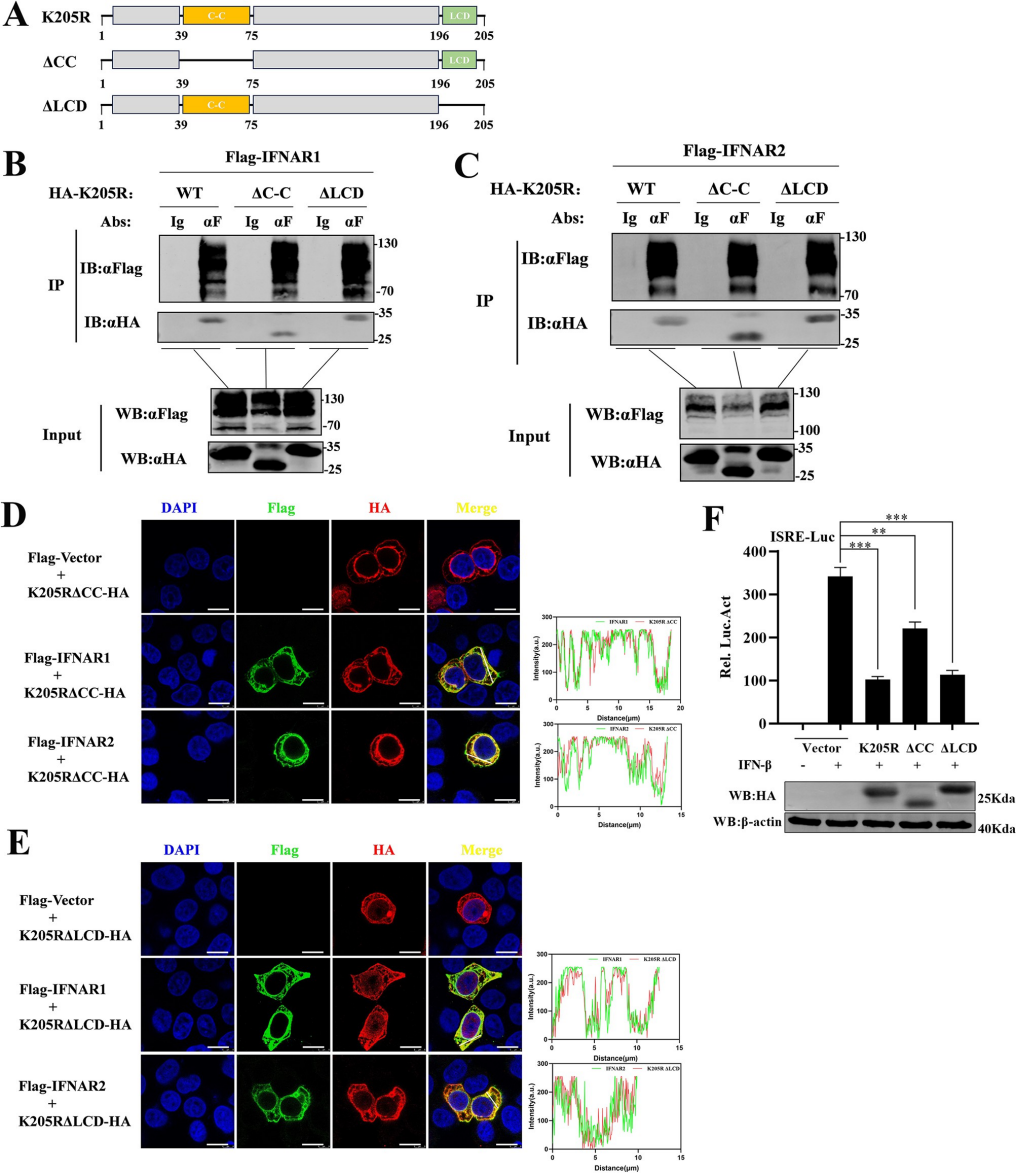
Identification of pK205R immunosuppressive functions of key domain structure. (A) Schematic representation of a deletion mutant constructed from the predicted pK205R functional domain. (B, C) HEK293T cells were co-transfected with pK205R (5 μg), PK205R-ΔCC (5 μg), and PK205R-ΔLCD (5 μg) along with IFNAR1 (5 μg) and IFNAR2 (5 μg) in 100-mm dishes. At 24 hpt, cell lysates were subjected to Co-IP using FLAG antibody, followed by immunoblotting with FLAG and HA. (D, E) HeLa cells were transfected with pK205R (1 μg), pK205R-ΔCC (1,000 ng), and pK205R-ΔLCD (1,000 ng) along with IFNAR1 (1 μg) and IFNAR2 (1 μg), alone or in combination. At 24 hpt, cells were stained with anti-FLAG (green) and anti-HA (red) antibodies, and the nucleus was stained with DAPI (blue). Colocalization of the indicated proteins was analyzed by confocal microscopy (scale bar: 10 μm). For the fluorescence intensity analysis, red and green wavy lines indicate fluorescence of the corresponding color. The ordinate represents the gray value of the fluorescence channel, and the abscissa represents the relative position of the lines in the merged plot. (F) HEK293T cells cultured in 24-well plates were co-transfected with pK205R (400 ng), pK205R-ΔCC (400 ng), or pK205R-ΔLCD (400 ng), along with PRL-TK (25 ng) and ISRE-Luc (125 ng). After 24 h, cells were treated with 1,000 U/mL IFN-β for 8 h and analyzed using a dual-luciferase reporter assay. ** P < 0.01; *** P < 0.001.

To define the functional domains of pK205R responsible for its immunosuppressive effects, we predicted the major domains of pK205R using Smart (https://smart.embl.de/), an online protein domain prediction tool, and constructed plasmids containing pK205R domain deletion mutants (Fig 6A). Subsequently, pK205R and its mutants (pK205R-ΔCC (coiled-coil) and pK205R-ΔLCD (low complexity domain)) were co-transfected into HEK293T cells with IFNAR1 and IFNAR2, respectively. Co-IP was used to detect the interaction between the pK205R mutants and IFNAR1/2. Unexpectedly, all pK205R mutants interacted with IFNAR1 and IFNAR2 (Fig 6B and 6C), as confirmed by confocal microscopy (Fig 6D and 6E).

We then assessed the effect of the pK205R mutants on IFN-I-activated ISRE promoter activity using a dual-luciferase reporter gene. We found that pK205R-ΔCC significantly attenuated the inhibitory effect on ISRE promoter activity compared to pK205R-WT. Despite this, pK205R-ΔCC still significantly inhibited IFN-I signaling (Fig 6F). Moreover, we found that the expression of pK205R-ΔCC was significantly lower than that of pK205R and pK205R-ΔLCD (S3 Fig). Therefore, the attenuated inhibitory effect of pK205R-ΔCC on IFN-I signaling may be attributed to its lower protein expression. This indicates that the CC domain is essential for the stability of pK205R. In conclusion, our findings suggest that the CC and LC domains of pK205R do not affect its antagonistic effect on IFN-I signaling.

### Multiple amino acid sites of pK205R mediate its interaction with IFNAR1/2

**Fig 7:**
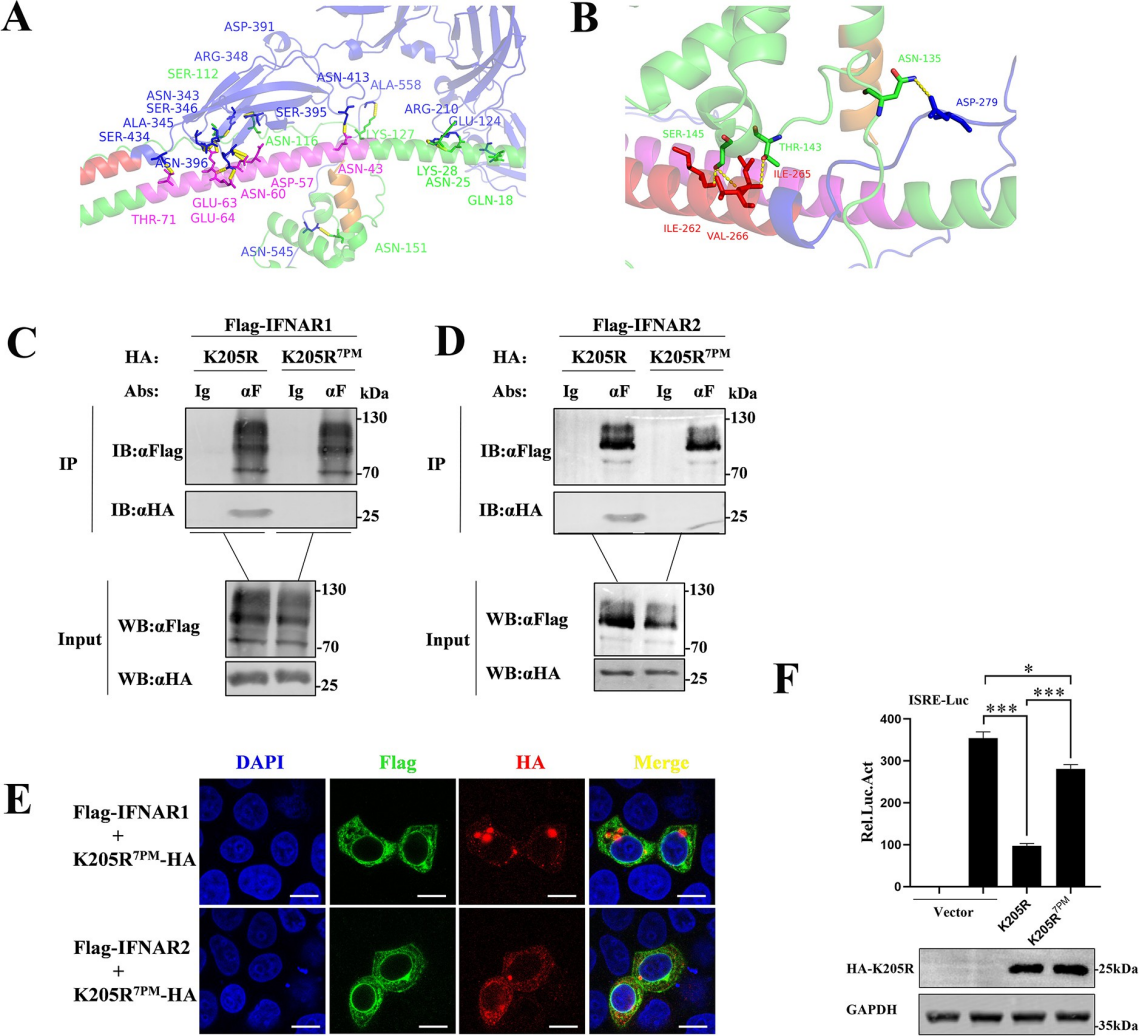
Identification of key amino acid sites mediating pK205R interaction with IFNAR1/2. (A, B) Docking model of pK205R with IFNAR1 and IFNAR2. IFNARs structure is shown in blue, TMD of IFNAR1 and IFNAR2 in red, pK205R structure in green, CCD in pink, and LCD domain in orange. (C, D) HEK293T cells in 100-mm culture dishes were co-transfected with pK205R (5 μg), pK205R^7PM^ (5 μg), and either IFNAR1 (5 μg) or IFNAR2 (5 μg). Cell lysates were subjected to Co-IP using FLAG antibody, followed by immunoblotting with FLAG and HA antibodies. (E) HeLa cells in glass-bottom dishes were transfected with pK205R (1 μg), pK205R^7PM^ (1 μg), and either IFNAR1 (1 μg) or IFNAR2 (1 μg), alone or in combination. At 24 hpt, cells were stained with anti-FLAG (green) and anti-HA (red) antibodies, and nuclei were stained with DAPI (blue). Colocalization of the indicated proteins was analyzed using confocal microscopy (scale bar: 10 μm). (F) HEK293T cells cultured in 24-well plates were co-transfected with pK205R (400 ng) or pK205R^7PM^ (400 ng), along with PRL-TK (25 ng) and ISRE-Luc (125 ng), respectively. After 24 h, cells were treated with 1,000 U/mL IFN-β for 8 h and analyzed using a dual-luciferase reporter assay. * P < 0.05; *** P < 0.001.

To further explore the mechanisms of pK205R interactions with IFNAR1 and IFNAR2, we used the Alphafold2 server (https://alphafold.ebi) to predict the structures of pK205R, IFNAR1, and IFNAR2, and the Gramm-x online software (http://gramm.compbio.ku.edu/) to construct docking models for pK205R-IFNAR1 and pK205R-IFNAR2. We analyzed the docking models, selecting the two with the highest scores as primary references (Fig 7A and 7B), with the remaining two docking models included in the supplementary material (S4A and S4B Fig). From the protein and docking model analysis, we identified seven amino acid residues in pK205R (Q18, N25, K32, N43, R47, T143, and S145) that may mediate interactions with important residues in IFNAR1/2. We mutated these residues to alanine, creating the pK205R point mutation expression vector pK205R^7PM^. Co-IP experiments showed that pK205R^7PM^ lost the ability to interact with IFNAR1 and IFNAR2 (Fig 7C and 7D). Consistent with these results, pK205R^7PM^ also lost the ability to co-localize with IFNAR1/2 (Fig 7E), although its subcellular localization was unchanged (S4C Fig). Using a dual-luciferase reporter assay, we found that pK205R^7PM^ greatly restored IFN-I signaling, though some inhibition of IFN-I signaling was still observed (Fig 7F). In conclusion, our data show that pK205R interacts with key amino acid sites in IFNAR1/2 and functions as a multifunctional immunosuppressive protein capable of targeted suppression of IFN-I signaling beyond IFNAR interactions.

### Construction of ASFV-pK205R^7PM^

**Fig 8:**
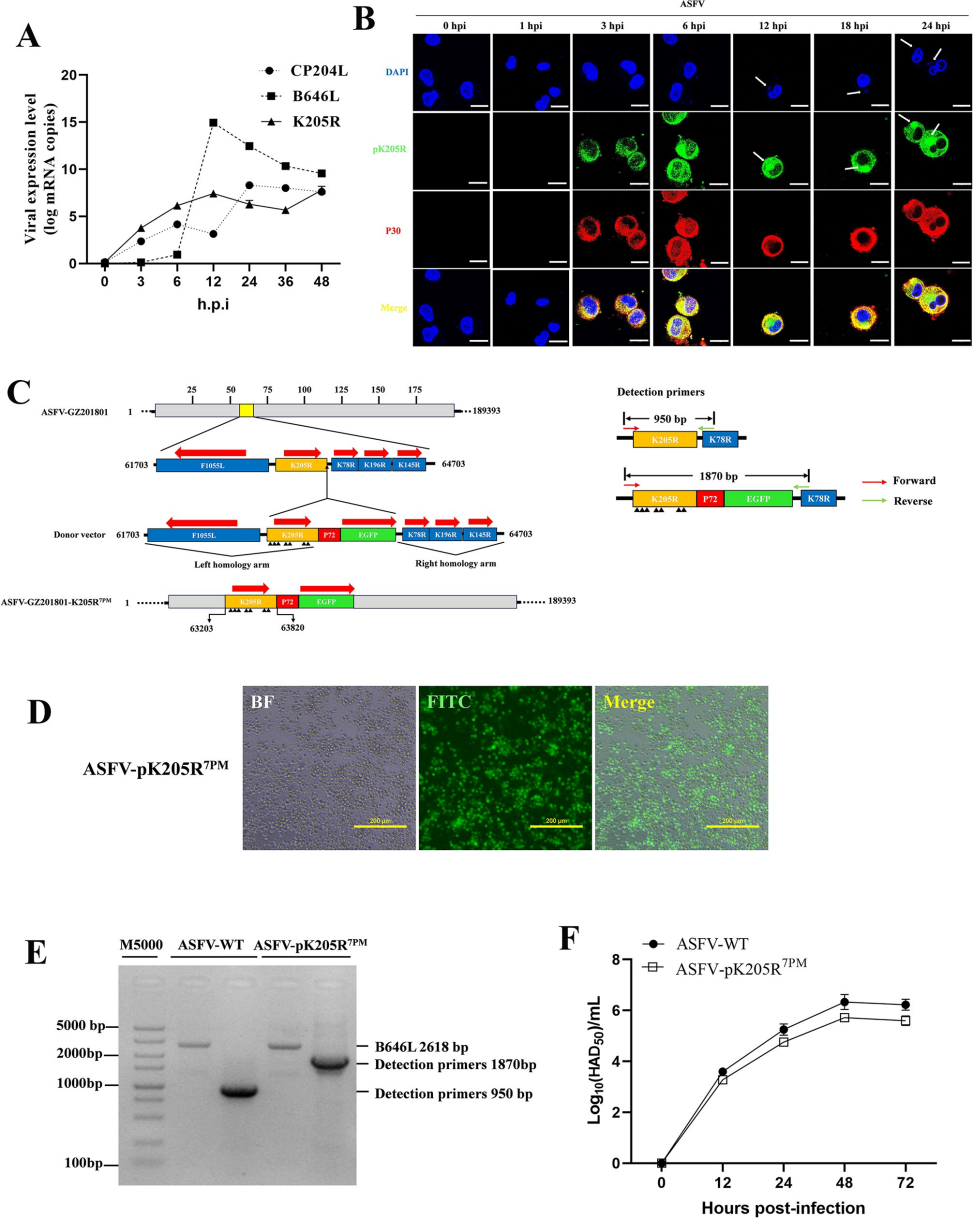
Construction of ASFV-pK205R^7PM^. (A) PAMs were infected with ASFV (MOI = 0.1), and cells were collected at the indicated time points post-infection. qPCR was used to detect the mRNA expression levels of K205R, CP204L, and B646L. (B) PAMs were infected with ASFV (MOI = 0.1), and cells were collected at the indicated time points post-infection. Cells were stained with anti-pK205R (green) and anti-p30 (red) antibodies, and nuclei were stained with DAPI (blue). Protein localization was analyzed by confocal microscopy (scale bar: 10 μm). (C) Schematic representation of ASFV-pK205R^7PM^ constructed by homologous recombination and schematic representation of detection primers, the location of the mutation site is marked with a black triangle. (D) Green fluorescence signal of purified ASFV-pK205R^7PM^ was observed under a fluorescence microscope. (E) Purity of recombinant strains was determined via PCR. Viral DNA obtained from the parental ASFV or ASFV-PK205R^7PM^ mutant was amplified via PCR using the K205R and B646L primers described above, with B646L serving as an indicator of genomic input. (F) PAMs were infected with ASFV-WT (MOI = 0.01) or ASFV-pK205R^7PM^ (MOI = 0.01), and the virus titers at the specified time points post-infection were detected using the HAD_50_ method. Growth curves of the two strains were plotted.

Next, we examined the transcriptional phase and subcellular localization of pK205R during ASFV infection at different time points. As expected, early ASFV infection led to significant pK205R transcription, even higher than CP204L (p30) at 12 h post-infection (hpi) (Fig 8A). Laser confocal microscopy revealed that both pK205R and p30 were detected at 3 hpi, with pK205R localizing to the virus factory by 12 hpi, co-localizing with p30 (Fig 8B). To further explore the immunosuppressive function of pK205R during ASFV infection, we attempted to construct ASFV-ΔK205R (S5A Fig). Despite the gradual decrease in wild-type virus with increasing purification passages (S5B Fig), we were unable to fully purify ASFV-ΔK205R (S5C Fig), indicating that pK205R is likely essential for virus replication, as it is involved in the virus factory during mid to late infection stages. Consequently, we constructed ASFV-pK205R^7PM^, designed detection primers (Fig 8C), and successfully purified ASFV-pK205R^7PM^ (Fig 8D and 8E). Sequencing of ASFV-pK205R^7PM^ and whole-genome NGS results of ASFV-pK205R^7PM^ are provided in the additional material (S5D and S5E Fig and S1 Dataset). Seven amino acids of pK205R were successfully mutated. Notably, proline at position 67 and 110 of pO174L were mutated to serine, and serine at position 75 was mutated to phenylalanine. The pO174L mutation may appears to have been introduced during the purification of ASFV-pK205R^7PM^, it does not affect the results of this study. Because the O174L gene deletion strain is not important for ASFV replication in vitro and in vivo or for disease production in domestic pigs [23]. Furthermore, our previous results showed that pO174L did not inhibit TBK1-activated ISRE promoter activity [20]. Then we plotted the growth curves of the two strains. The growth curve of ASFV-pK205R^7PM^ was slightly lower than that of ASFV-WT (Fig 8F), which may be attributed to its reduced immunosuppressive capacity. To investigate this, we tested the sensitivity of ASFV-WT and ASFV-pK205R^7PM^ to IFN-β. Compared to ASFV-WT, ASFV-pK205R^7PM^ was more sensitive to IFN-β, confirming our hypothesis (S6A Fig). Additionally, Co-IP was performed to analyze the interaction of pK205R with endogenous IFNAR1 and IFNAR2 in PAMs infected with ASFV or ASFV-pK205R^7PM^. pK205R interacted with endogenous IFNAR1 and IFNAR2 in ASFV-WT-infected PAMs but not in ASFV-pK205R^7PM^-infected PAMs (S6B Fig).

### ASFV pK205R^7PM^ restored IFN-I signal transduction

**Fig 9:**
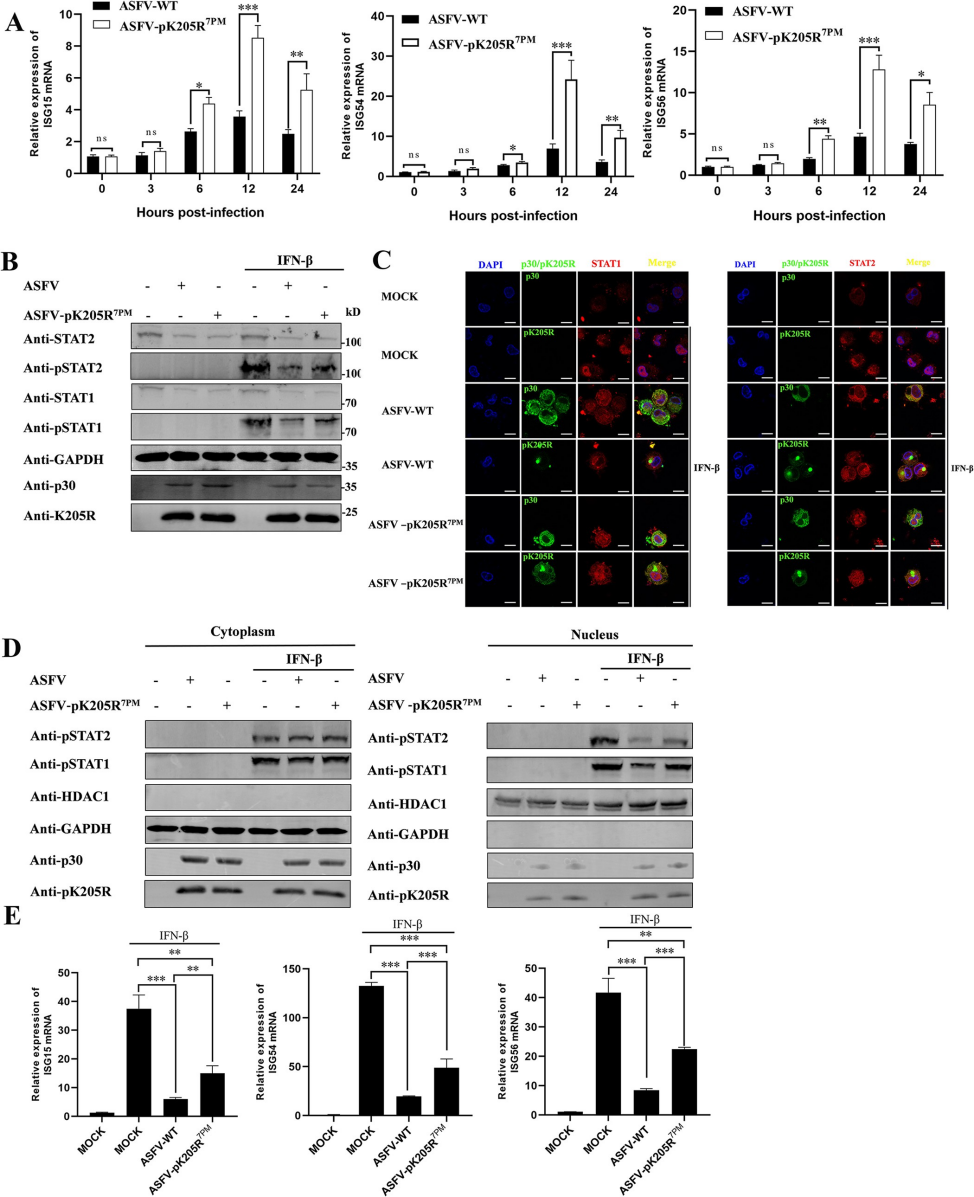
Effect of ASFV-WT and ASFV-pK205R^7PM^ on IFN-I signaling. (A) PAMs were infected with ASFV-WT or ASFV-pK205R^7PM^ (MOI = 0.1), and cells were harvested at the indicated time points for determination of ISG15, ISG54, and ISG56 mRNA levels. (B–D) PAMs were infected with ASFV-WT or ASFV-pK205R^7PM^ (MOI = 0.1). At 24 hpi, cells were stimulated with IFN-β (1,000 U/mL) for 2 h. (B) Protein immunoblotting was performed using indicated antibodies. (C) Cells were stained with anti-pK205R or p30 (green) and anti-STAT1/2 (red) antibodies, and nuclei were stained with DAPI (blue). Protein localization was analyzed by confocal microscopy (scale bar: 10 μm). (D) p-STAT1/2 levels in nuclear and cytoplasmic compartments were detected using western blotting. GAPDH and heat shock protein HDAC1 were used as cytoplasmic and nuclear markers, respectively. (E) PAMs were infected with ASFV-WT or ASFV-pK205R^7PM^ (MOI = 1) for 24 h and then treated with IFN-β (1,000 U/ml) for 8 h. mRNA levels of ISG15, ISG54, and ISG56 were determined using RT-qPCR. ns, P > 0.05; * P < 0.05; ** P < 0.01; *** P < 0.001.

To further confirm that pK205R antagonizes IFN-I signaling through the IFNAR during ASFV infection, we monitored the transcriptional levels of ISG15, ISG54, and ISG56 induced by ASFV-WT and ASFV-pK205R^7PM^ at different time points of infection with PAMs. The results are shown in Fig 9A. Compared to ASFV-WT, the transcription of ISG15, ISG54, and ISG56 induced by ASFV-pK205R^7PM^ was significantly increased from 6 hpi. Further investigation revealed that ASFV-pK205R^7PM^ partially restored STAT1 and STAT2 phosphorylation (Figs 9B and S7A) and attenuated the inhibitory effect on STAT1/p-STAT1 and STAT2/p-STAT2 nuclear translocation compared to ASFV-WT (Figs 9C and 9D and S7B). Finally, we assessed the differences in exogenous IFN-I activated ISGs between ASFV-WT and ASFV-pK205R^7PM^. ASFV-pK205R^7PM^ showed reduced inhibition of IFN-I activated ISG transcription compared to ASFV-WT (Fig 9E). Taken together, our data indicate that ASFV-pK205R^7PM^ induces higher levels of ISGs and alleviates, but does not completely abolish, the inhibitory effect on IFN-I signaling compared with ASFV-WT. This is because ASFV encodes various proteins targeting different nodes of the IFN-I pathway as a compensatory mechanism to antagonize IFN-I signaling [24]. Notably, CD2v, pH240R, and pB318L also target IFNAR to inhibit IFN-I signaling [25–27]. Therefore, we assessed whether these three proteins and pK205R had additive effects on IFN-I inhibition. We found that only pB318L, but not pH240R or CD2v, cooperated with pK205R to inhibit IFN-I signaling (S8 Fig).

## Discussion

IFN-I is the first line of defense against viruses and can induce a variety of antiviral cytokines to inhibit viral replication at all stages of the viral life cycle [28,29]. Despite this, ASFV has evolved various immune escape pathways to inhibit IFN-I innate immunity and promote its own replication. Moreover, the immune escape and pathogenic mechanisms of ASFV have not been fully elucidated. Although ASFV utilizes a variety of proteins to target upstream IFN-I to block IFN-I production [17,18], some IFN-I can still be detected upon ASFV infection, thereby activating IFN-I downstream signaling [4,30]. Therefore, investigating how ASFV overcomes IFN-I-triggered defenses is crucial for understanding ASFV-mediated immune escape and developing new prevention strategies. Our data indicate that ASFV pK205R antagonizes IFN-I signaling by inhibiting the binding of JAK1-IFNAR2 and TYK2-IFNAR1 through interactions with the ICDs of IFNAR1 and IFNAR2. Importantly, the pK205R^7PM^ mutant lost its ability to interact with IFNAR1 and IFNAR2, significantly weakening the inhibitory effect on IFN-I signaling both as a single protein and during viral infection. Given that pK205R is an early protein and that IFNARs are initiation proteins that determine the intensity of IFN-I signaling [8], we hypothesized that pK205R targets IFNAR to inhibit IFN-I signaling and disrupt the host immune system response early in ASFV infection. This disruption lays the groundwork for efficient replication in the middle and late stages of infection. In conclusion, our results reveal the immunosuppressive effects of ASFV pK205R.

IFNAR serves as a critical element in initiating IFN-I signaling. Knockout mouse models lacking key genes in the IFN-I pathway, such as IRF3^−/−^, MAVs^−/−^, demonstrate reduced sensitivity to ZIKV, while those lacking IRF3^−/−^, IRF5^−/−^, IRF7^−/−^, and IFNAR1^−/−^ exhibit minimal resistance to ZIKV challenge [31]. This highlights the importance of IFNAR for IFN-I in establishing a collective antiviral state against pathogens. Consequently, viruses employ various mechanisms to antagonize IFN-I signaling through IFNAR. For instance, vaccinia virus produces a soluble protein, B18R, which mimics IFNAR1, thus limiting IFN-I binding to IFNAR and dampening IFN-I signaling [32]. Similarly, Kaposi’s sarcoma-associated herpesvirus RIF protein inhibits IFN-I signaling by inhibiting IFNAR binding to JAKs [33], and herpes simplex virus 1 achieves a similar effect by disrupting IFNAR2 binding to JAK1 [34]. Additionally, ASFV CD2v, pB318L, and pH240R have been reported to inhibit IFN-I signaling by inhibiting IFNAR binding to JAKs [25–27]. Given the important role of IFNAR in IFN-I signaling, we explored the mechanism by which the novel ASFV immunosuppressive protein pK205R targets IFNAR to suppress host innate immunity.

**Fig 10:**
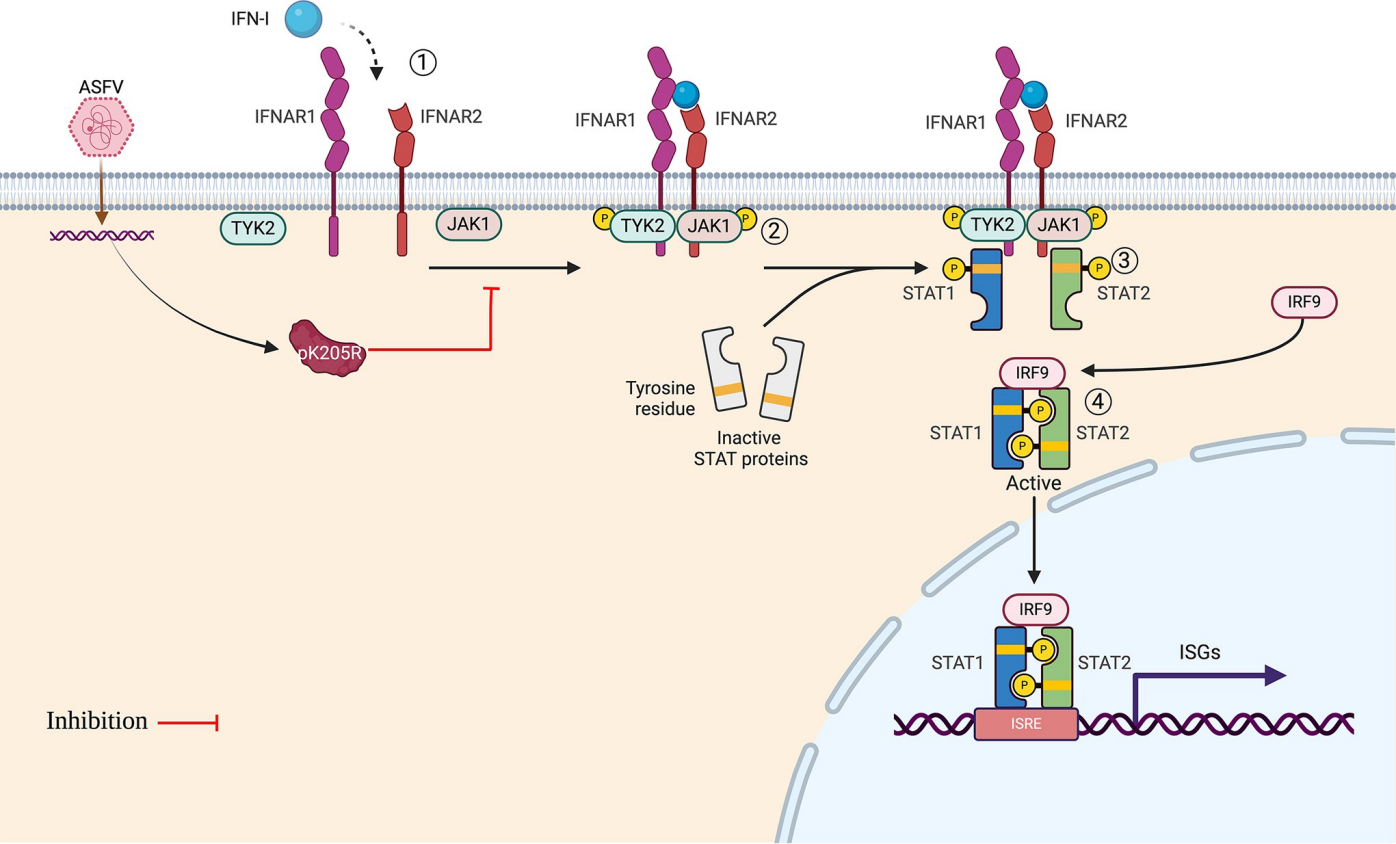
ASFV pK205R antagonism of IFN-I signaling. ASFV pK205R targets IFNAR1 and IFNAR2, inhibiting the interaction between IFNAR1-TYK2 and IFNAR2-JAK1, thereby blocking downstream signaling pathways. Created with Biorender.com.

Our experiments using truncated receptor subunits and truncated and mutant forms of pK205R revealed the details of the molecular mechanisms by which pK205R blocks IFN-I signaling through the receptor. The IFNAR ECD is responsible for the interaction with IFN-I. The ECD of the high-affinity subunit IFNAR2 exhibits NMR structural features comprising two fibronectin type III (FNIII)-like domains (D1 and D2) [35], whereas the ECD of the low-affinity subunit IFNAR1, unique to class II helical cytokine receptors, consists of four FNIII-like domains (SD1–SD4). Among these, SD4, proven unnecessary for IFN-I binding [11]. Our results suggest that pK205R interacted with both ICD and ECD of IFNAR1 and IFNAR2, but mainly with ICD. ICD of IFNARs is associated with the phosphokinases TYK2 and JAK1. The Box1 motif of IFNARs is mainly responsible for the regulation and activation of JAK1 and TYK2, while the Box2 motif is responsible for binding JAK1 and TYK2 [36,37]. Our data indicate that pK205R strongly interacts with the ICD of IFNAR1 and IFNAR2. Upon ligand binding, IFNAR cross-phosphorylates TYK2 and JAK1, leading to phosphorylation of tyrosine residues on IFNAR ICD and members of the STAT family. Phosphorylated STATs translocate to the nucleus and drive ISG transcription [7,10]. Combining these findings with our results, we can infer that pK205R targets the ICDs of IFNARs, inhibiting IFNAR1-TYK2 and IFNAR2-JAK1 interactions. Consequently, this leads to reduced JAK phosphorylation and STAT activation, inhibiting STAT nuclear translocation and hindering IFN-I signal transduction (Fig 10).

The generation of ASFV immunosuppression gene recombinant strains hold promise for effective ASFV vaccine research and development, a significant step forward in combating the disease [38,39]. Although we encountered challenges in constructing an ASFV-ΔK205R mutant strain, our failure to do so underscores the importance of pK205R in ASFV replication. We speculate that the presence of homologous arms in the recombinant virus may facilitate nucleic acid release during replication, leading to recombination with wild-type virus. To address this, we synthesized the pK205R^7PM^ plasmid and constructed an ASFV-pK205R^7PM^ point-mutant recombinant strain. Both pK205R^7PM^ and ASFV-pK205R^7PM^ showed reduced inhibition of IFN-I signaling in HEK293T cells and porcine alveolar macrophages (PAMs). However, ASFV-pK205R^7PM^ did not fully restore IFN-I signaling, likely due to the presence of multiple ASFV proteins inhibiting signaling at different points in the JAK/STAT pathway. For example, pMGF360-9L degrades STAT1 and STAT2 [40], while pI215L degrades IRF9 through autophagy [41]. Hence, ASFV employs multiple immunosuppressive proteins to ensure its replication, acting as a backup mechanism to counteract host innate immunity.

In conclusion, we explored a novel mechanism by which ASFV evades IFN-I antiviral responses. pK205R targets IFNAR1 and IFNAR2 to inhibit IFN-I signaling. In addition, we present a new ASFV recombinant strain, advancing our understanding of ASFV–host interactions and facilitating the development of ASFV vaccines.

## Materials and methods

### Antibodies and reagents

IRF9 polyclonal antibody (pAb) (14167–1—AP), STAT2 pAb (16674–1—AP), STAT1 pAb (10144–2—AP), GFP tag monoclonal antibody (mAb) (66002–1—Ig), JAK1 mAb (66466–1—Ig), TYK2 mAb (67411–1—Ig), GAPDH mAb (60004–1—Ig), HA-Tag mAb (66006–2—Ig), and DYKDDDDK Tag mAb (20543–1—AP) were purchased from Proteintech (Wuhan China). IFNAR1 mAb (ab124764) was purchased from Abcam, whereas IFNAR2 mAb (#53883), HA-Tag Rabbit mAb (# 3724), FLAG-Tag Rabbit mAb (# 14793), Phospho-Jak1 (Tyr1034/1035) Rabbit mAb (D7N4Z; #74129), Phospho-Tyk2 (Tyr1054/1055) Rabbit mAb (#68790), IRF9 Rabbit mAb (#76684), Phospho-Stat1 (Tyr701) Rabbit mAb (#9167), and Phospho-Stat2 (Tyr690) Rabbit mAb (#88410) were purchased from Cell Signaling Technology. The pK205R pAb was a generous gift from Professor Peng Gao of the China Agricultural University, pK205R rabbit pAb and pK205R mouse mAb were generously donated by ProtTech Inc (Luo Yan, China). p30 mAb was prepared and stored in the laboratory.

Protein A/G Plus-agarose (sc-2003) was purchased from Santa Cruz Biotechnology. Dulbecco’s Modified Eagle Medium (DMEM) high sugar, RPMI 1640 Medium, Opti—MEM Medium, NE-PER (#78833), FastDigest Kpn I (FD0524), FastDigest EcoR I (FD0274), FastDigest Sac I (FD1134), and FastDigest Xho I (FD0694) were purchased from Thermo Fisher Scientific Inc. Alexa Fluor 488-labeled Goat Anti-Mouse IgG(H+L) (A0428), Alexa Fluor 647-labeled Goat Anti-Rabbit IgG(H+L) (A0468), and Alexa Fluor 555-labeled Donkey Anti-Mouse IgG(H+L) (A0460) were purchased from Biyuntian. The dual-luciferase reporter gene assay kit (11402ES60) was purchased from Yeasen Biotechnology (Shanghai, China). Recombinanti Human IFN-β (300-02BC) was purchased from Peputec Biotech (Suzhou, China) Co., LTD. Goat Anti-Mouse IgG H&L (IRDye 800 CW) (926–32210) and Goat Anti-Rabbit IgG H&L (IRDye 800 CW) (925–32211) secondary antibodies were purchased from LI-COR, USA.

### Plasmids

pRL-TK, pISRE-Luc, pCAGGS-K205R-HA, pCAGGS-K205R-FLAG, pEGFP-C1-K205R, pCDNA3.1–3×FLAG-STAT1, pCDNA3.1–3×FLAG-STAT2, pCDNA3.1–3×FLAG-IRF9, and pBlueScript SK (+) were prepared in our laboratory. PCAGGS-HA-IFNAR1, PCAGGS-HA-IFNAR2, PCAGGS-FLAG-IFNAR1, and PCAGGS-FLAG-IFNAR2 were generously donated by Professor Weng Changjiang of the Harbin Veterinary Research Institute. pCDNA3.1–3×FLAG JAK1 and pCDNA3.1–3×FLAG TYK2 were constructed via reverse transcription of total RNA extracted from PAMs, followed by PCR amplification and cloning into pCDNA3.1–3× FLAG. PCAGGS-FLAG-IFNAR1-ΔECD, PCAGGS-FLAG-IFNAR1-ΔICD, PCAGGS-FLAG-IFNAR2-ΔECD, and PCAGGS-FLAG-IFNAR2-ΔICD were cloned into PCAGGS-FLAG using PCAGGS-HA-IFNAR1 and PCAGGS-HA-IFNAR2 as templates. Additionally, PCAGGS-FLAG-IFNAR1-ECD, PCAGGS-FLAG-IFNAR1-ICD, PCAGGS-FLAG-IFNAR2-ECD, and PCAGGS-FLAG-IFNAR2-ICD were cloned using PCAGGS-HA-IFNAR1 and PCAGGS-HA-IFNAR2 as templates. PCAGGS-K205R-ΔCC and PCAGGS-K205R-ΔLCD were cloned into PCAGGS-HA using ASFV GZ201801 cDNA as a template. PCAGGS-HA-K205R^7PM^ was synthesized by Sangon Biological Co., Ltd. All plasmids were confirmed through sequencing. The primer sequences used in this study are listed in S1 Table.

### Viruses and cell culture

ASFV GZ201801 (GenBank: MT496893.1) and GFP were maintained in our laboratory. HEK293T and HeLa cells were cultured in DMEM containing 10% fetal bovine serum (FBS) and 1% penicillin/streptomycin. PAMs were maintained in our laboratory and cultured in RPMI 1640 medium supplemented with 10% FBS and 1% penicillin/streptomycin.

### Luciferase assay

HEK293T cells were co-transfected with pRL-TK, ISRE-luc, or the indicated plasmids. After 12 h of transfection, the cells were stimulated with or without 1,000 U/mL IFN-β for 8 h, and a negative control group was set up. The cells were then washed once with pre-cooled sterile PBS. ISRE and TK activities were detected using a dual-luciferase reporter gene assay kit according to the manufacturer’s instructions. The ISRE fluorescence activity/TK fluorescence activity value was used for the analysis.

### Extraction of total cellular RNA using qPCR

Total cellular RNA was extracted according to the instructions of the Total RNA Rapid Extraction Kit (Feijie, Shanghai, China) and reverse transcribed using the HiScript II 1st Strand cDNA Synthesis Kit (+gDNA wiper). The resulting cDNA was analyzed by qPCR using a Roche LightCycler 480 II qPCR instrument with 2 × ChamQ SYBR qPCR Master Mix (Without ROX), performing each sample in triplicate. All samples were normalized to the GAPDH transcript levels. Primer sequences used in this study are listed in S2 Table.

### Western blot

The cells were lysed with cell lysate, centrifuged at 4°C, and the supernatant was collected. Samples tested for endogenous IFNAR were ultrasonically fragmented, reducing the protein lysate amount by a factor of one. Subsequently, 5× protein loading buffer was added, and the proteins were denatured by mixing and boiling for 10 min. The protein samples were stored at –20°C after transient centrifugation. A 10% SDS-PAGE protein gel was prepared according to the instructions of the PAGE Rapid Gel configuration kit (Yase). Protein markers and samples were added to the wells in sequence and the electrophoresis program was set at 80 V for 20 min, then 120 V for 1 h. The gel was transferred to a methanol-activated PVDF membrane, assembled in a transfer sandwich (sponge, filter paper, gel, PVDF membrane, filter paper, sponge) and placed in a transfer tank with ice, applying a constant current of 200 mA for membrane transfer. This was followed by blocking with 5% skim milk powder for 2 h. Primary antibodies were diluted in proportion with 2% BSA and incubated overnight at 4°C. Secondary antibodies were diluted in 5% skimmed milk and incubated for 1 h at room temperature in the dark. Images were acquired using an Odyssey infrared dual-channel laser scanning imaging system (LI-COR) and photographed.

### Co-IP

The specified plasmids were transfected into HEK293T cells. After transfection, the cells were lysed with Western and IP lysis buffers containing PMSF on ice for 10 minutes. The lysate was centrifuged at 15,000 × *g* for 10 min at 4° C, and 100 μL of the supernatant was reserved as input protein samples. The remaining supernatant was purified with 20 μL of protein A/G agarose and 5 μL of IgG homologous to the IP antibody for 12 h, incubating at 4°C for 12 h. Post-incubation, the samples were centrifuged at 1,000 × *g* for 3 min at 4°C. The purified supernatant was divided equally into IP antibody tubes and IgG tubes, followed by a 2-h incubation at 4°C. The supernatant was discarded, and the pellets were washed three times with 1 mL PBST, each wash involving a 5-min incubation at 4°C and centrifugation at 1,000 × *g* for 3 min at 4°C. After the final wash, 50 μL of 1× protein sample buffer was added to the protein A/G agarose, and the mixture was boiled for 8 min to denature the proteins. The samples were then centrifuged at 12,000 × *g* for 2 min to obtain the IP Protein samples. Subsequently, western blotting was performed to analyze the results.

### Indirect immunofluorescence assay and confocal microscopy

The indicated plasmids were transfected into HEK293T cells. The cells were washed twice with pre-cooled PBS, fixed with 4% paraformaldehyde for 10 min at room temperature, and permeabilized with 2 mL of 0.3% Triton X-100 for 10 min at room temperature. Blocking was performed with 5% BSA (prepared in PBS) for 1 h at 37° C. Primary antibodies, prepared in PBS, were incubated overnight at 4°C, followed by two washes with pre-cooled PBS. Secondary antibodies, also prepared in PBS, were incubated for 1 h at 37°C in the dark. Cells were then stained with DAPI for 5 min at 37°C. The results were observed and analyzed using a Zeiss LSM710 laser confocal scanning microscope.

### Construction of the ASFV gene deletion strain

ASFV-pK205R^7PM^ was constructed via homologous recombination using ASFV GZ201801 as the backbone. The ASFV P72 promoter fragment was combined with the EGFP fragment and 1.5-kb upstream and downstream sequences of the K205R stop codon. The K205R ORF fragment in the homology arm was constructed using the pK205R^7PM^ plasmid as the template, and the DNA of ASFV-GZ201801 was used as the template for the rest of the homology arms. The above DNA fragments were cloned into the empty PBSK vector to form the ASFV-pK205R^7PM-^EGFP homologous arm donor plasmid screening expression cassette. This PBSK-K205R^7PM^-EGFP construct was transfected into PAMs using TransIT-LT1 Transfection Reagent (MIR 2300), and inoculated with ASFV GZ201801 4 h later. Green fluorescence was observed 2 d later, and the rescued virus was collected and purified by limited-fold dilution. Viral titers were determined using the HAD_50_ assay [42]. The primer sequences used for vector construction are listed in S1 Table, the detection primers are shown in S3 Table.

### Cell cytotoxicity assay

CCK8 assay was used to detect the cytotoxicity of the transfected plasmids. Briefly, HEK293T was dispensed into 96-well plates, followed by plasmid transfection. The assay was performed in triplicates, including a blank control. At 24 hpt, CCK8 was added and incubated for 1 h. Absorbance was then measured at 450 nm. Cytotoxicity was calculated using Equation “1- (OD transfection/OD blank)”, The results are presented in S9 Fig.

### Statistical analysis

Statistical significance was assessed via a two-tailed Student’s t-test using GraphPad Prism (version 8; San Diego, California, USA). *, P < 0.05, **, P < 0.01, and ***, P < 0.001 were considered statistically significant.

## Supporting information

S1 Fig Densitometry of the target protein bands shown in Fig 2 and calculation of their relative densitometric ratios to GAPDH, STAT1, STAT2, or HDAC1. (A, B, C) Densitometry of the target protein bands shown in Fig 2A–2C and calculation of their relative densitometric ratios to GAPDH, STAT1, or STAT2. (D) Percentage of cells exhibiting nuclear localization of STAT1 and STAT2 in Fig 2D. (E) Densitometry of the target protein bands shown in Fig 2E and calculation of their relative densitometric ratios to GAPDH or HDAC1. Data represent the mean ± SD of three independent experiments. (ns, no significance; ** p < 0.01).
(TIF)

S2 Fig Interaction of pK205R with key proteins of the JAK/STAT pathway and subcellular localization of pK205R. (A) HEK293T cells were co-transfected with pK205R (5 μg) and JAK1/TYK2/STAT1/STAT2/IRF9 (5 μg) in 100-mm dishes. Co-IP of cell lysates was performed using HA and FLAG antibodies at 24 hpt, followed by protein immunoblotting using FLAG, HA, and EGFP antibodies. (B) pK205R (2 μg) was transfected into HeLa cells in glass-bottom dishes. At 24 hpt, the cells were stained with anti-HA (red), and the nucleus was stained with DAPI (blue). Colocalization of the indicated proteins was analyzed using confocal microscopy (scale bar: 10 μm).
(TIF)

S3 Fig Densitometry of the pK205R bands and calculation of their relative densitometric ratios to GAPDH. HEK293T cells were transfected with K205R, K205R-ΔCC, or K205R-ΔLCD in 24-well plates. At 24 hpt, the expression of the indicated proteins was determined by western blotting.
(TIF)

S4 Fig Identification of key amino acid sites mediating the interaction between pK205R and IFNAR1/2, along with the subcellular localization of pK205R^7PM^. (A, B) Docking model of pK205R with IFNAR1 and IFNAR2. IFNARs structure is shown in blue, TMD of IFNAR1 and IFNAR2 in red, pK205R structure in green, CCD in pink, and LCD domain in orange. (C) pK205R^7PM^ (2 μg) was transfected into HeLa cells in glass-bottom dishes. At 24 hpt, the cells were stained with anti-HA (red) antibodies, and the nuclei were stained with DAPI (blue). Colocalization of the indicated proteins was analyzed using confocal microscopy (scale bar: 10 μm).
(TIF)

S5 Fig Construction of ASFV-ΔK205R. (A) Schematic representation of homologous recombination for constructing ASFV-ΔK205R. (B) ASFV-ΔK205R was purified by limited-fold dilution and treated with porcine erythrocytes from PAMs infected with ASFV-ΔK205R. (C) Purity assessment of ASFV-ΔK205R strains via PCR detection. Viral DNA obtained from the parental ASFV-WT or ASFV-ΔK205R was PCR amplified using K205R and B646L primers (B646L primer served as an indicator of genomic input). (D) Sequencing results of ASFV-pK205R^7PM^. (E) Partial sequence alignment results after whole genome sequencing of ASFV-WT and ASFV-pK205R^7PM^. The results of whole-genome sequencing are provided in S1 Dataset.
(TIF)

S6 Fig Sensitivity test of ASFV-WT and ASFV-pK205R^7PM^ to IFN-β and Interaction of ASFV-WT and ASFV-pK205R^7PM^ with IFNAR1 and IFNAR2. (A) PAMs were infected with ASFV-WT (MOI = 0.1) or ASFV-pK205R^7PM^ (MOI = 0.1). At 3 hpi, PAMs were treated with IFN-β (10 U/mL 100 U/mL, 1000 U/mL). At 24 hpi, the cells were stained with anti-p30 (red) and pK205R (green) antibodies, and the nuclei were stained with DAPI (blue) (scale bar: 100 μm). (B) PAMs were infected with ASFV-WT or ASFV-pK205R^7PM^ in 100-mm dishes. Co-IP of cell lysates was performed using mouse pK205R antibodies at 24 hpi, followed by immunoblotting using rabbit pK205R, IFNAR1, and IFNAR2 antibodies.
(TIF)

S7 Fig Densitometry of the target protein bands shown in Fig 9 and calculation of their relative densitometric ratios to GAPDH, HDAC1, STAT1, or STAT2. (A) Densitometry of the target protein bands shown in Fig 9B and calculation of their relative densitometric ratios to STAT1, or STAT2. (B) Densitometry of the target protein bands shown in Fig 9D and calculation of their relative densitometric ratios to GAPDH or HDAC1.
(TIF)

S8 Fig The inhibitory effects of pB318L and pK205R on IFN-I signal transduction are additive. HEK293T cells cultured in 24-well plates were transfected with K205R expression plasmid or empty vector (150 ng), PRL-TK (25 ng), and ISRE-Luc (125 ng). CD2v, H240R, and B318L expression plasmids were transfected with K205R alone or in combination. The cells were treated with 1000 U/mL IFN-β for 8 h at 24 hpt, and cell viability was assessed using the dual luciferase reporter gene assay.
(TIF)

S9 Fig Cytotoxicity assay of the plasmids used in this study. HEK293T cells cultured in 96-well plates were transfected with the expression plasmid or empty vector used in this study, respectively. At 24 hpt, HEK293T cell viability was analyzed by Cell Counting Kit-8 assay.
(TIF)

S1 Table The primers used for cloning. (XLSX)

S2 Table The primers used for quantitative real-time PCR. (XLSX)

S3 Table The primers used for real-time PCR. (XLSX)

S1 Dataset Whole genome sequencing of ASFV-pK205R^7PM^. (DNA). (RAR)

S2 Dataset Excel spreadsheet containing, in separate sheets, the underlying numerical data and statistical analysis graph for Figs 1A, 1B, 6F, 7F, 9A, 9E, S1A, S1B, S1C, S1D, S1E, S7A, S7B, S8 and S9. (RAR)
