# Supplementary figures and images for: pK205R targets the proximal element of IFN-I signaling pathway to assist African swine fever virus to escape host innate immunity at the early stage of infection
Source: PLoS Pathog. 2024 Oct 15;20(10):e1012613. doi: 10.1371/journal.ppat.1012613 (PMC11508493; doi:10.1371/journal.ppat.1012613)

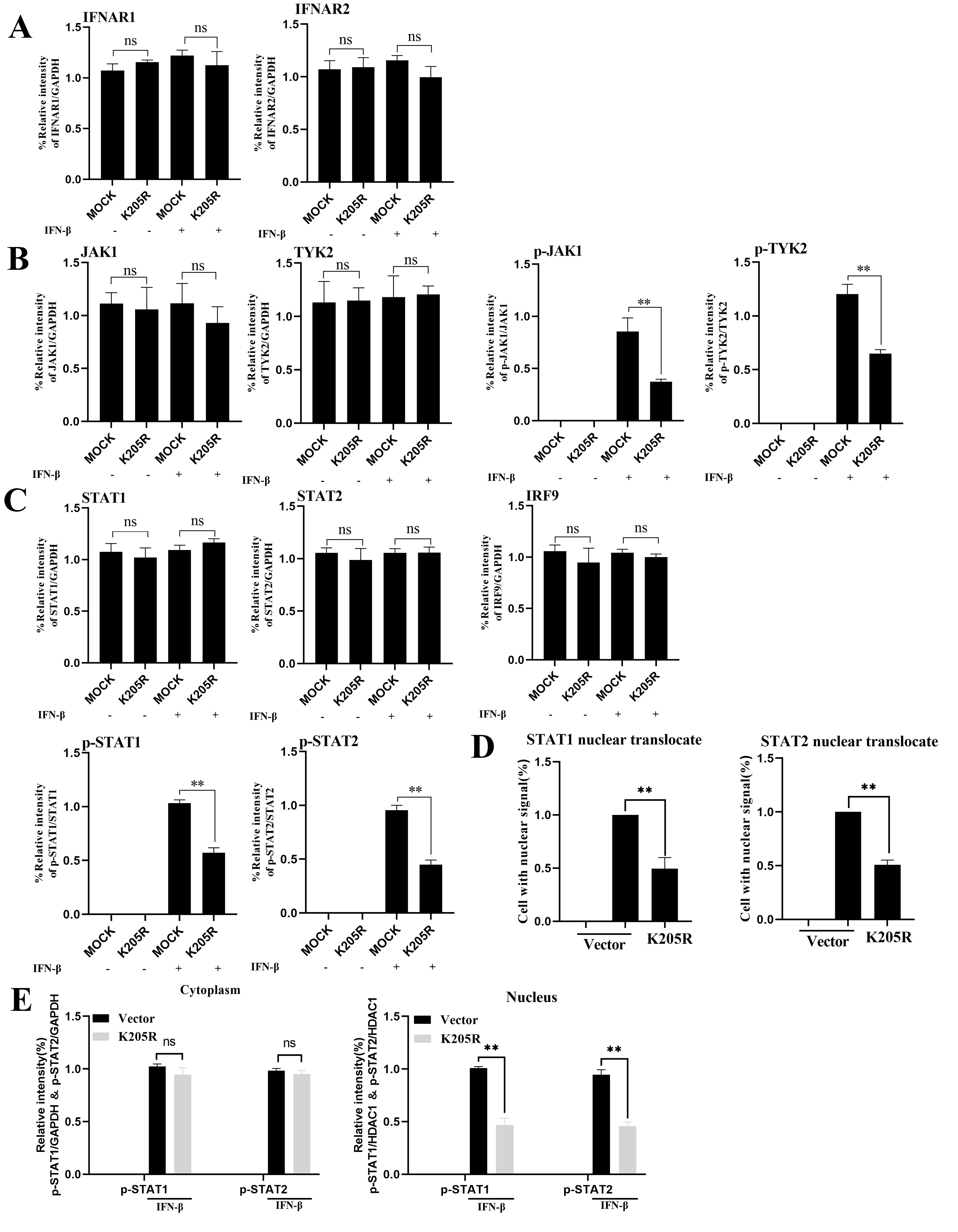

Supplement: S1 Fig — (A, B, C) Densitometry of the target protein bands shown in Fig 2A–2C and calculation of their relative densitometric ratios to GAPDH, STAT1, or STAT2. (D) Percentage of cells exhibiting nuclear localization of STAT1 and STAT2 in Fig 2D. (E) Densitometry of the target protein bands shown in Fig 2E and calculation of their relative densitometric ratios to GAPDH or HDAC1. Data represent the mean ± SD of three independent experiments. (ns, no significance; ** p < 0.01). (TIF) [file ppat.1012613.s001.tif]

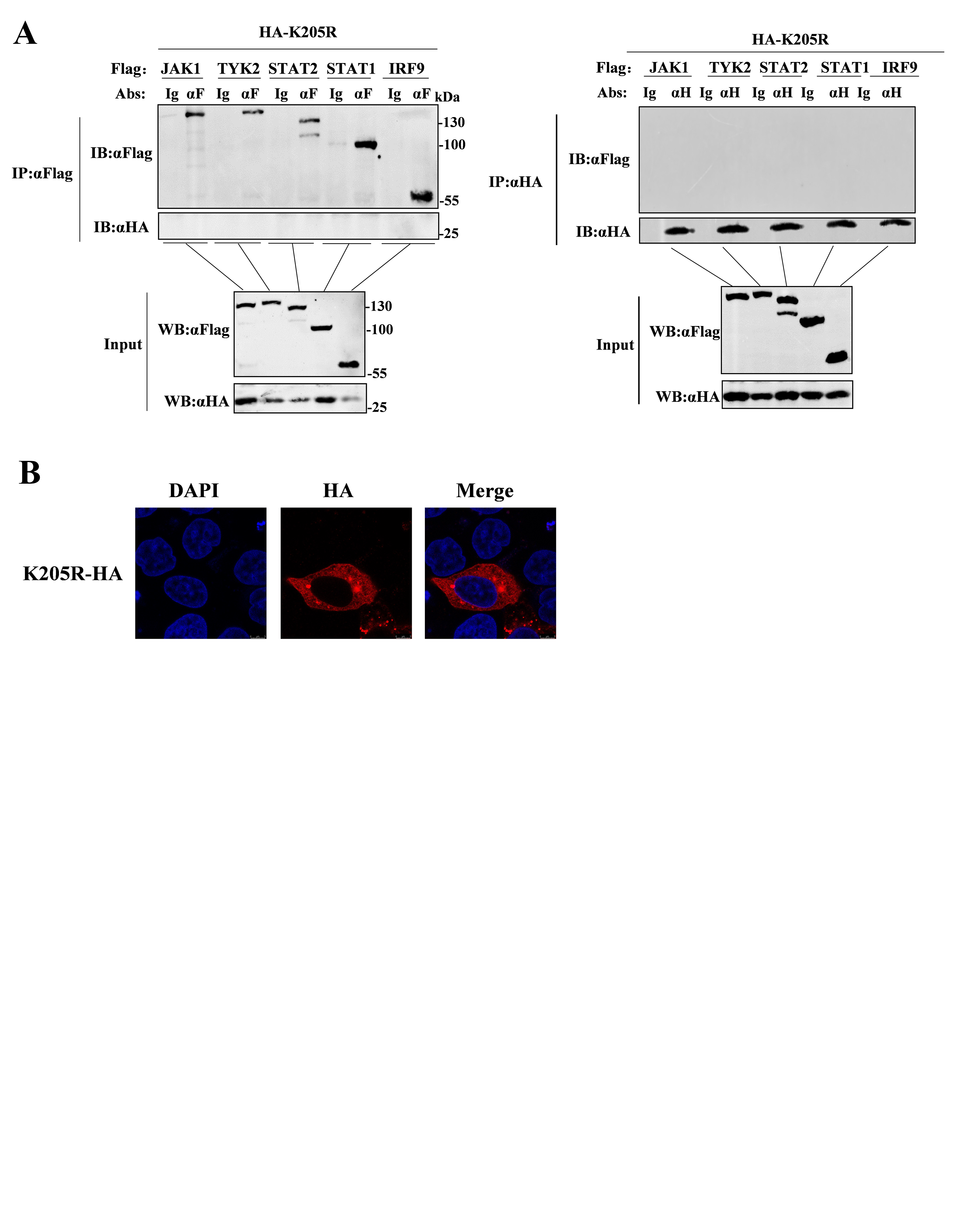

Supplement: S2 Fig — (A) HEK293T cells were co-transfected with pK205R (5 μg) and JAK1/TYK2/STAT1/STAT2/IRF9 (5 μg) in 100-mm dishes. Co-IP of cell lysates was performed using HA and FLAG antibodies at 24 hpt, followed by protein immunoblotting using FLAG, HA, and EGFP antibodies. (B) pK205R (2 μg) was transfected into HeLa cells in glass-bottom dishes. At 24 hpt, the cells were stained with anti-HA (red), and the nucleus was stained with DAPI (blue). Colocalization of the indicated proteins was analyzed using confocal microscopy (scale bar: 10 μm). (TIF) [file ppat.1012613.s002.tif]

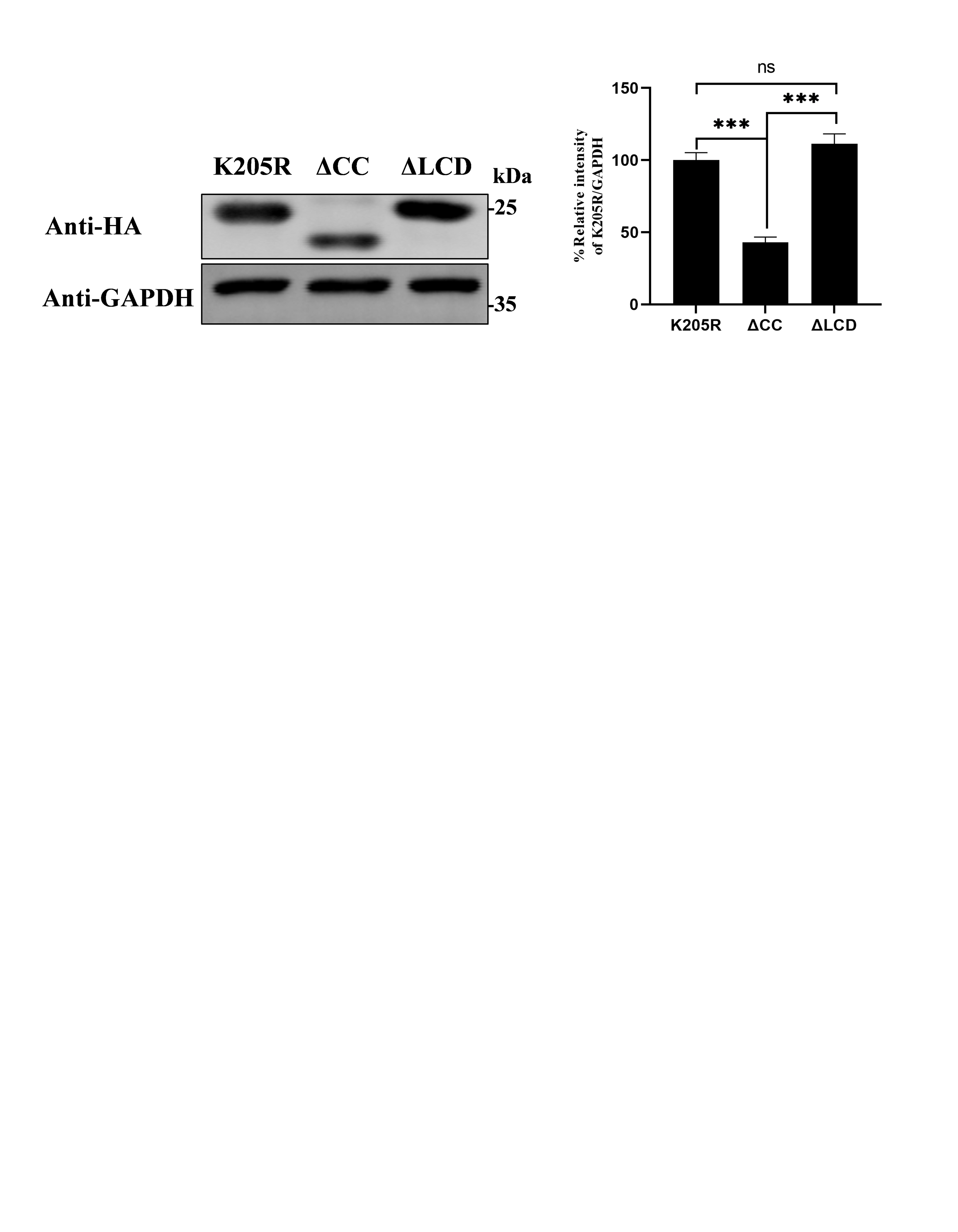

Supplement: S3 Fig — HEK293T cells were transfected with K205R, K205R-ΔCC, or K205R-ΔLCD in 24-well plates. At 24 hpt, the expression of the indicated proteins was determined by western blotting. (TIF) [file ppat.1012613.s003.tif]

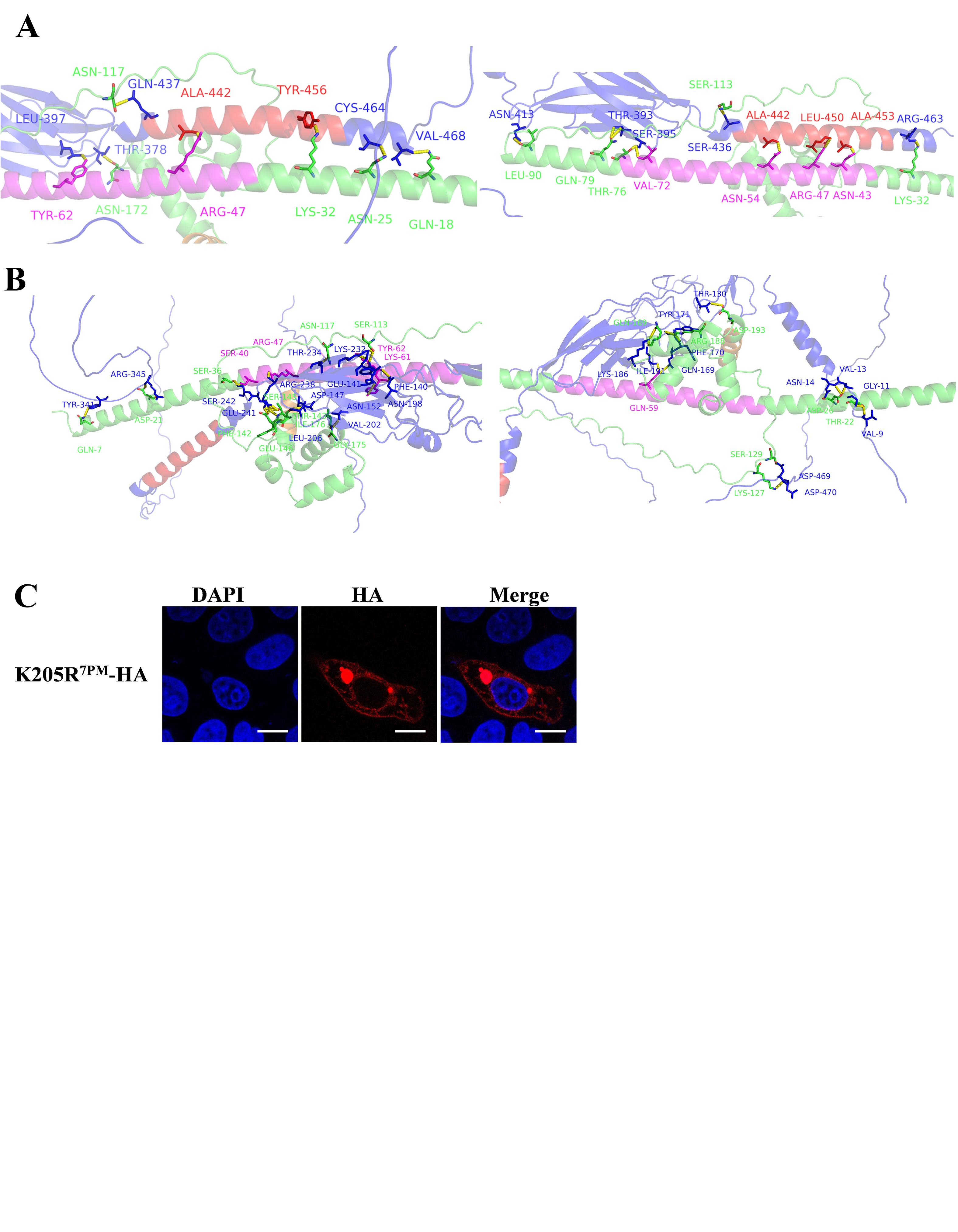

Supplement: S4 Fig — (A, B) Docking model of pK205R with IFNAR1 and IFNAR2. IFNARs structure is shown in blue, TMD of IFNAR1 and IFNAR2 in red, pK205R structure in green, CCD in pink, and LCD domain in orange. (C) pK205R7PM (2 μg) was transfected into HeLa cells in glass-bottom dishes. At 24 hpt, the cells were stained with anti-HA (red) antibodies, and the nuclei were stained with DAPI (blue). Colocalization of the indicated proteins was analyzed using confocal microscopy (scale bar: 10 μm). (TIF) [file ppat.1012613.s004.tif]

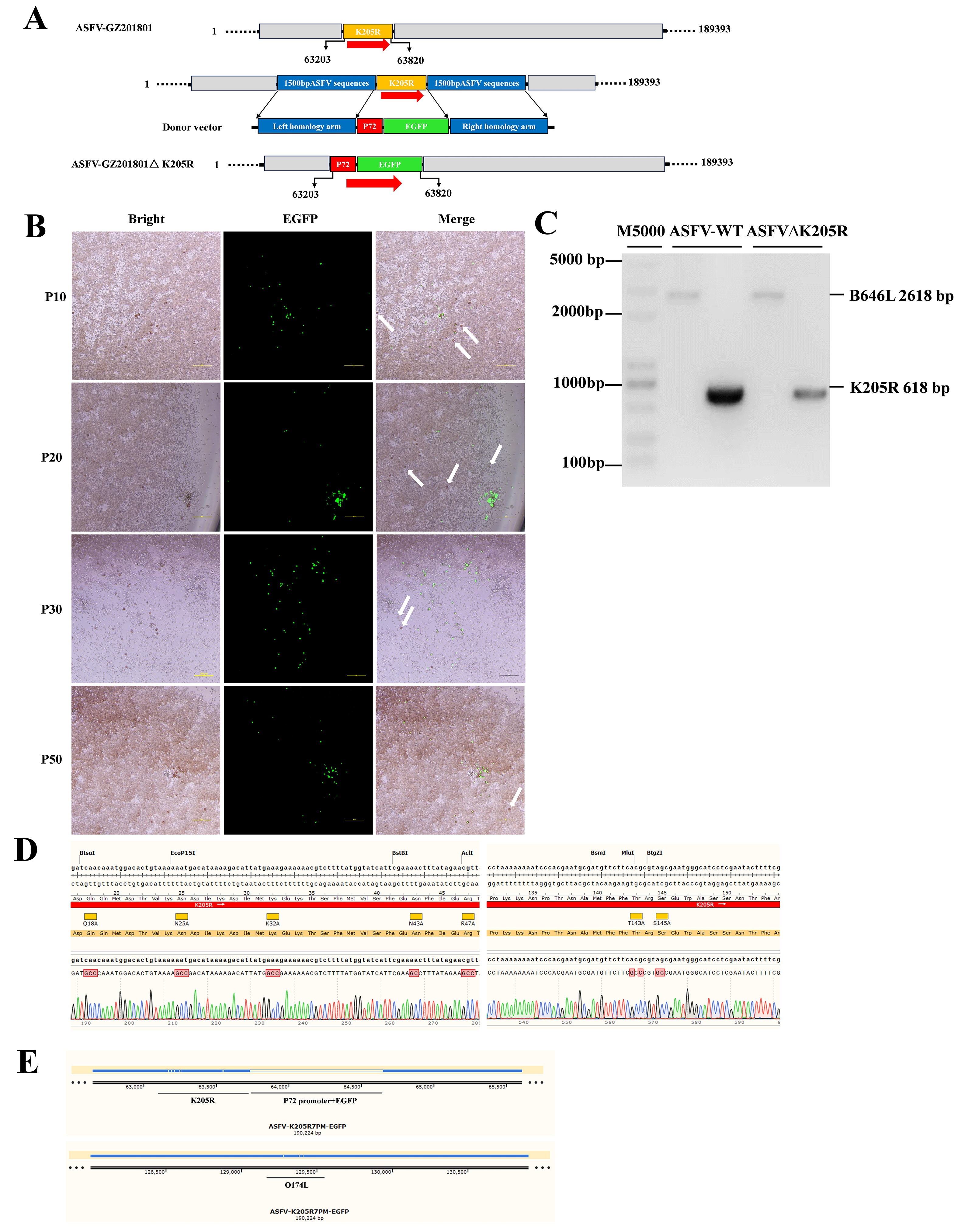

Supplement: S5 Fig — (A) Schematic representation of homologous recombination for constructing ASFV-ΔK205R. (B) ASFV-ΔK205R was purified by limited-fold dilution and treated with porcine erythrocytes from PAMs infected with ASFV-ΔK205R. (C) Purity assessment of ASFV-ΔK205R strains via PCR detection. Viral DNA obtained from the parental ASFV-WT or ASFV-ΔK205R was PCR amplified using K205R and B646L primers (B646L primer served as an indicator of genomic input). (D) Sequencing results of ASFV-pK205R7PM. (E) Partial sequence alignment results after whole genome sequencing of ASFV-WT and ASFV-pK205R7PM. The results of whole-genome sequencing are provided in S1 Dataset. (TIF) [file ppat.1012613.s005.tif]

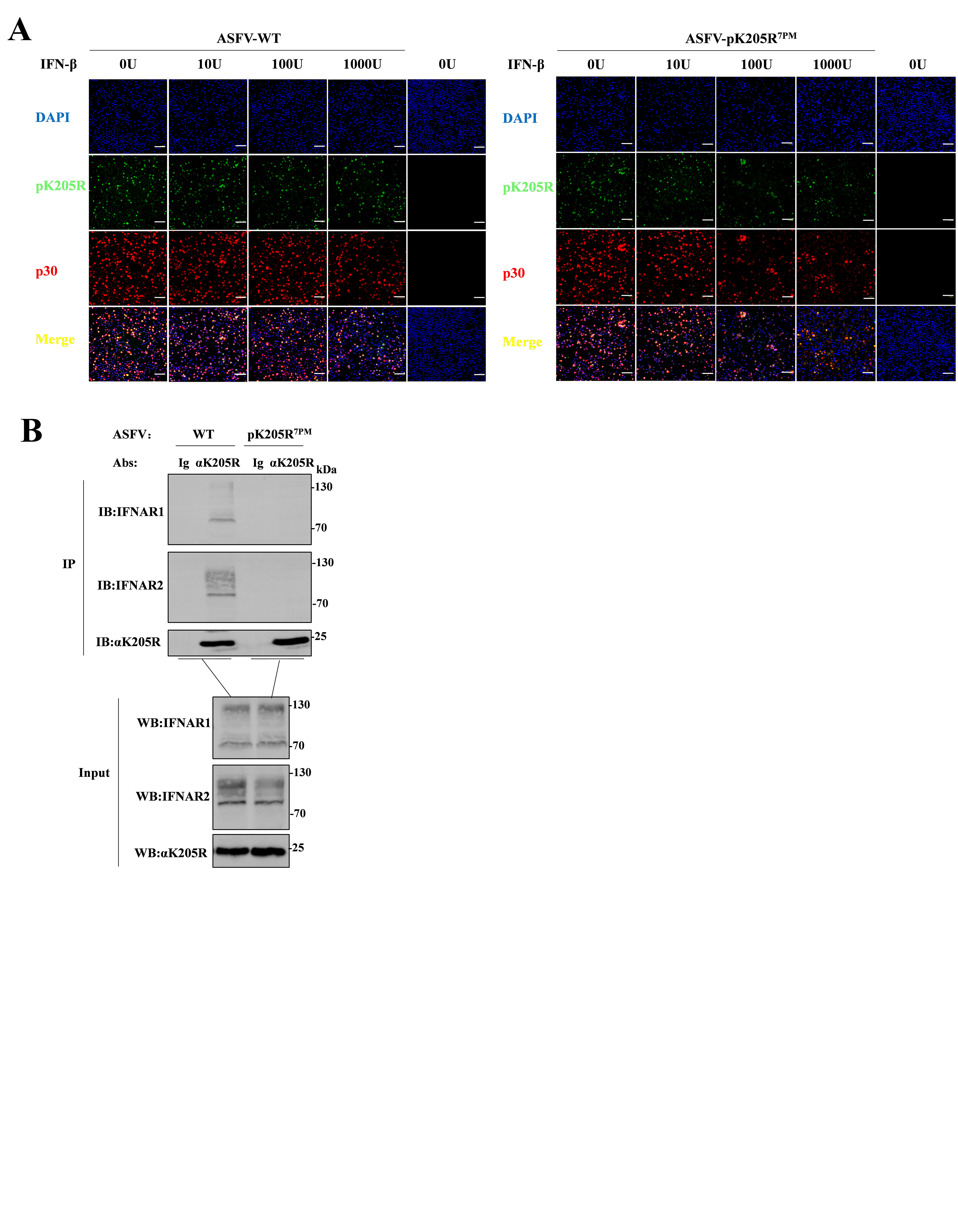

Supplement: S6 Fig — (A) PAMs were infected with ASFV-WT (MOI = 0.1) or ASFV-pK205R7PM (MOI = 0.1). At 3 hpi, PAMs were treated with IFN-β (10 U/mL 100 U/mL, 1000 U/mL). At 24 hpi, the cells were stained with anti-p30 (red) and pK205R (green) antibodies, and the nuclei were stained with DAPI (blue) (scale bar: 100 μm). (B) PAMs were infected with ASFV-WT or ASFV-pK205R7PM in 100-mm dishes. Co-IP of cell lysates was performed using mouse pK205R antibodies at 24 hpi, followed by immunoblotting using rabbit pK205R, IFNAR1, and IFNAR2 antibodies. (TIF) [file ppat.1012613.s006.tif]

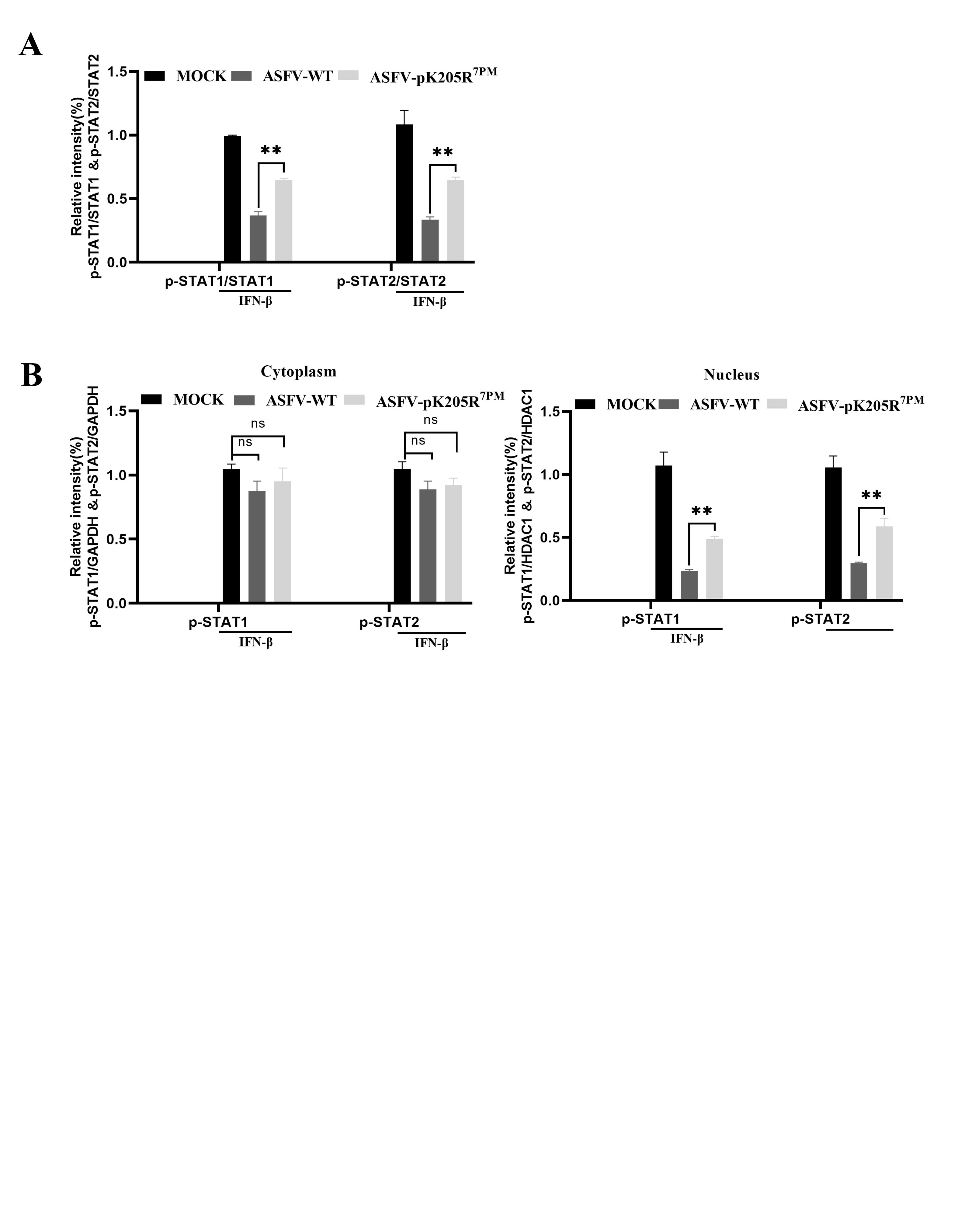

Supplement: S7 Fig — (A) Densitometry of the target protein bands shown in Fig 9B and calculation of their relative densitometric ratios to STAT1, or STAT2. (B) Densitometry of the target protein bands shown in Fig 9D and calculation of their relative densitometric ratios to GAPDH or HDAC1. (TIF) [file ppat.1012613.s007.tif]

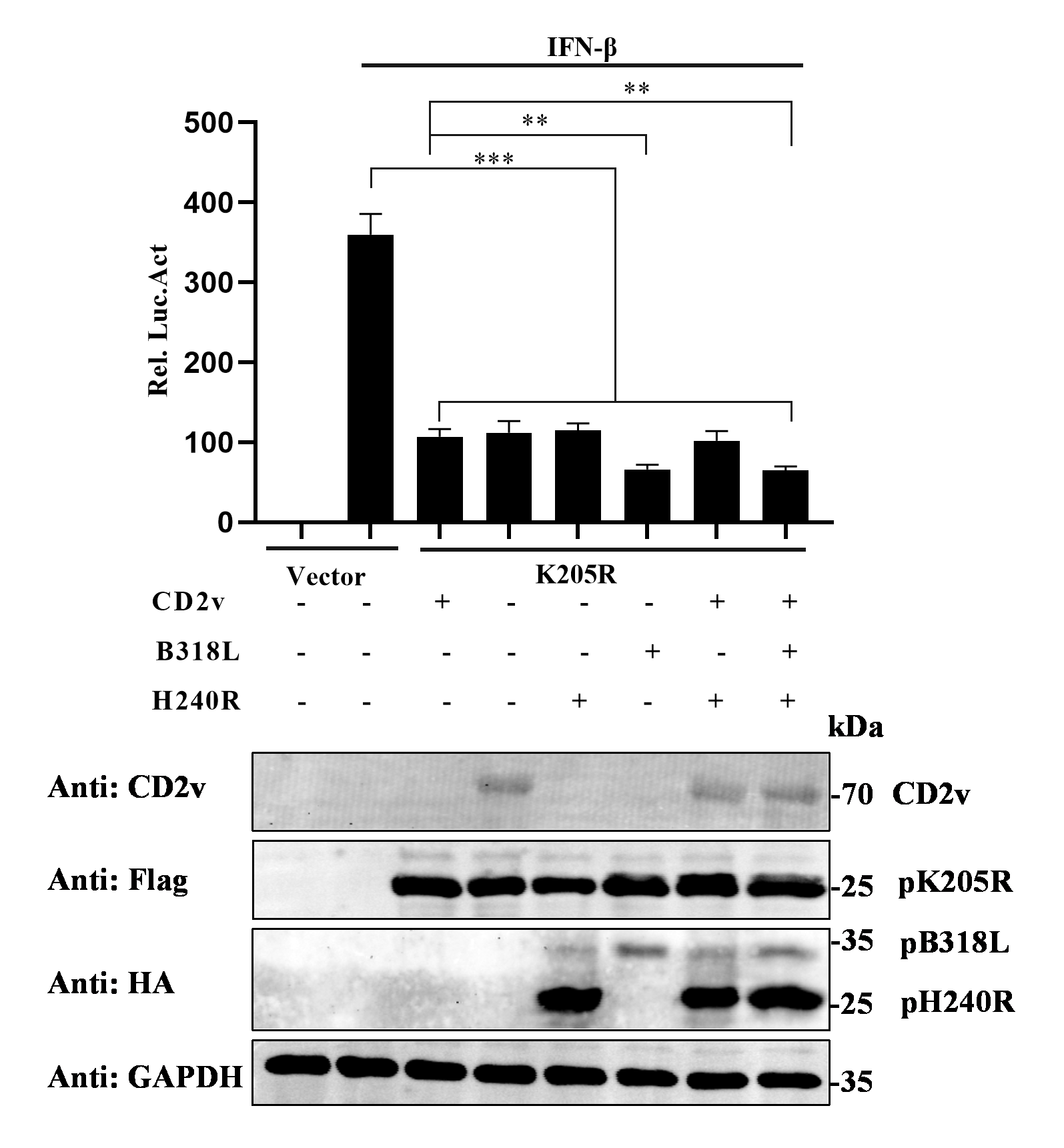

Supplement: S8 Fig — HEK293T cells cultured in 24-well plates were transfected with K205R expression plasmid or empty vector (150 ng), PRL-TK (25 ng), and ISRE-Luc (125 ng). CD2v, H240R, and B318L expression plasmids were transfected with K205R alone or in combination. The cells were treated with 1000 U/mL IFN-β for 8 h at 24 hpt, and cell viability was assessed using the dual luciferase reporter gene assay. (TIF) [file ppat.1012613.s008.tif]

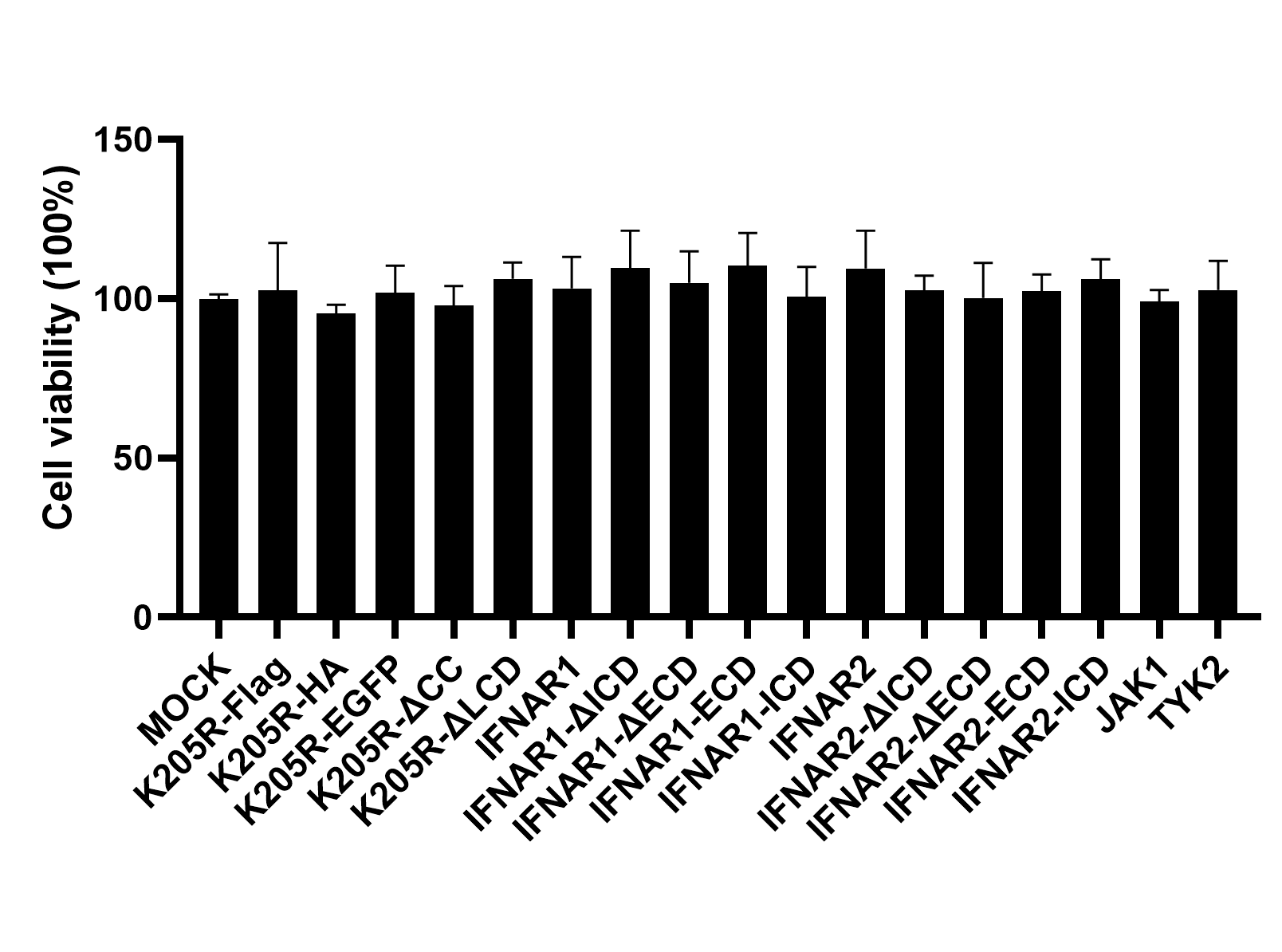

Supplement: S9 Fig — HEK293T cells cultured in 96-well plates were transfected with the expression plasmid or empty vector used in this study, respectively. At 24 hpt, HEK293T cell viability was analyzed by Cell Counting Kit-8 assay. (TIF) [file ppat.1012613.s009.tif]
